# Myeloid-associated differentiation marker is an essential host factor for human parechovirus PeV-A3 entry

**DOI:** 10.1038/s41467-023-37399-8

**Published:** 2023-03-31

**Authors:** Kanako Watanabe, Tomoichiro Oka, Hirotaka Takagi, Sergei Anisimov, Shun-ichi Yamashita, Yoshinori Katsuragi, Masahiko Takahashi, Masaya Higuchi, Tomotake Kanki, Akihiko Saitoh, Masahiro Fujii

**Affiliations:** 1 Division of Laboratory Science, Niigata University Graduate School of Health Sciences, Niigata, Japan; 2 Department of Virology II, National Institute of Infectious Diseases, Tokyo, Japan; 3 Management Department of Biosafety, Laboratory Animal, and Pathogen Bank, National Institute of Infectious Diseases, Tokyo, Japan; 4 Division of Virology, Niigata University Graduate School of Medical and Dental Sciences, Niigata, Japan; 5 Department of Cellular Physiology, Niigata University Graduate School of Medical and Dental Sciences, Niigata, Japan; 6 Faculty of Nursing, Niigata College of Nursing, Niigata, Japan; 7 Department of Microbiology, Kanazawa Medical University School of Medicine, Ishikawa, Japan; 8 Department of Pediatrics, Niigata University Graduate School of Medical and Dental Sciences, Niigata, Japan

**Keywords:** Viral infection, Virus-host interactions

## Abstract

Human parechovirus (PeV-A) is an RNA virus that belongs to the family *Picornaviridae* and it is currently classified into 19 genotypes. PeV-As usually cause mild illness in children and adults. Among the genotypes, PeV-A3 can cause severe diseases in neonates and young infants, resulting in neurological sequelae and death. In this study, we identify the human myeloid-associated differentiation marker (MYADM) as an essential host factor for the entry of six PeV-As (PeV-A1 to PeV-A6), including PeV-A3. The infection of six PeV-As (PeV-A1 to PeV-A6) to human cells is abolished by knocking out the expression of MYADM. Hamster BHK-21 cells are resistant to PeV-A infection, but the expression of human MYADM in BHK-21 confers PeV-A infection and viral production. Furthermore, VP0 capsid protein of PeV-A3 interacts with one extracellular domain of human MYADM on the cell membrane of BHK-21. The identification of MYADM as an essential entry factor for PeV-As infection is expected to advance our understanding of the pathogenesis of PeV-As.

## Abstract (web summary)

Human parechovirus genotype A3 (PeV-A3) can cause severe diseases in neonates and young infants. Here, Watanabe et al. identify a host factor myeloid-associated differentiation marker (MYADM) as essential for PeV-A infection and provide evidence for its involvement during virus entry into host cells in vitro.

## Introduction

Human parechovirus (PeV-A) is a non-enveloped, single-stranded, positive-sense RNA virus that belongs to the *Picornaviridae* family^1^. PeV-A is currently classified into 19 genotypes based on the VP1 sequences of the PeV-As. PeV-A infection usually causes mild respiratory and gastrointestinal disease in children and adults. Notably, infection with PeV-A3 sometimes causes serious disease in neonates and young infants, such as sepsis and meningoencephalitis^2^, which can result in severe neurological sequelae and death^3–5^. For the last two decades, epidemics of severe PeV-A3-related disease in neonates and young infants in the summer and autumn seasons have been regularly reported in different parts of the world^6–9^. Interestingly, PeV-As other than PeV-A3 are much less frequently associated with these serious infections in comparison to PeV-A3^1,2^.

The interaction of viral proteins with cell membrane receptors is an essential step in the viral infection of a host cell. Viral receptors are important determinants of viral etiologies, including tissue tropism, cytotoxicity and pathogenesis^10^.

In this study, we use a genome-wide gene-knockout strategy in PeV-A3-susceptible cells to identify human myeloid-associated differentiation marker (MYADM), a multi-membrane spanning protein, as a host factor essential for PeV-A3 entry and infection. The expression of human MYADM in hamster cells (BHK-21), which are not susceptible to PeV-A3 infection, induces productive infection with PeV-A3. Human MYADM binds to the VP0 capsid protein of PeV-A3, and the fourth extracellular region of MYADM is essential for the binding of MYADM with VP0 and productive infection of PeV-A3. In addition, besides PeV-A3, at least five other PeV-A genotypes use MYADM as an essential host factor for infection. The identification of human MYADM as a factor required for the infection of six PeV-As is therefore expected to facilitate the overall understanding of the pathogenesis of PeV-A infection.

## Results

### Human MYADM was isolated as a protein essential for PeV-A3 infection

**Fig. 1:**
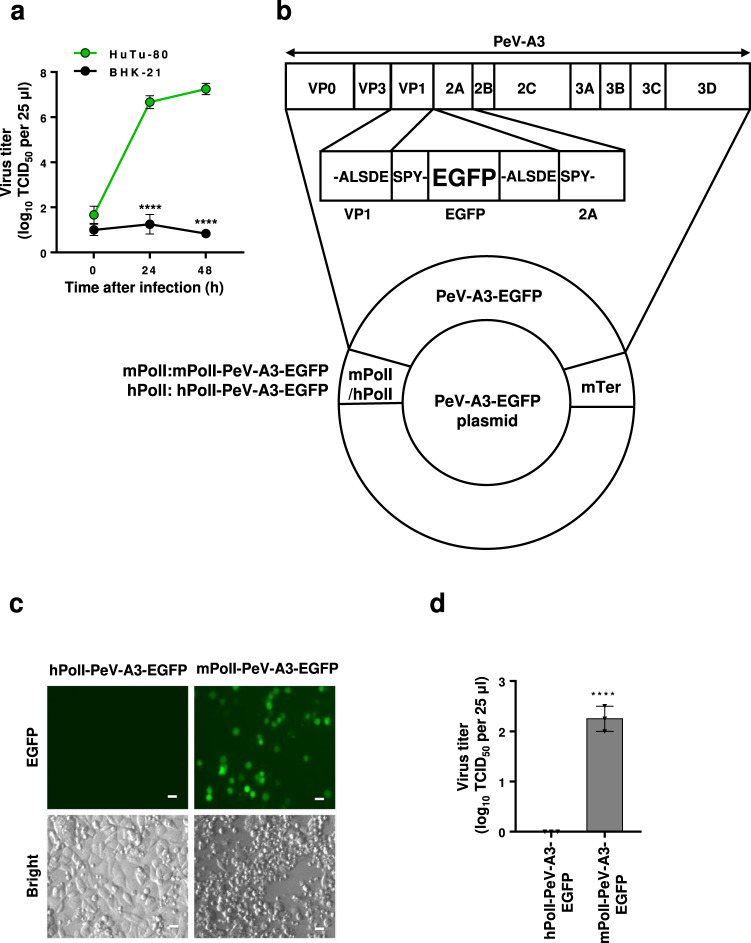
PeV-A3 does not infect hamster BHK-21 cells. **a** BHK-21 (black circle) and HuTu-80 (green circle) cells were infected with PeV-A3 (A308/99) at an MOI of 0.01, and the amount of virus (virus titer) in the culture supernatant was measured at 0, 24 and 48 h after infection. The virus titers in the culture supernatant of infected cells are shown as the mean and the standard deviation (s.d.) from triplicate experiments. Data from one experiment representative of two independent experiments (*N* = 2) are shown. *P* values were calculated by a two-way ANOVA with Sidak’s multiple comparisons test. ****P < 0.0001. **b** The hPolI-PeV-A3-EGFP and mPolI-PeV-A3-EGFP plasmids contain human (hPolI) and mouse (mPolI) RNA polymerase I promoters in addition to PeV-A3-EGFP DNA, respectively, and also have a transcription terminator DNA (mTer) of the mouse ribosomal gene. The EGFP in PeV-A3-EGFP has a PeV-A3 3C protease recognition cleavage peptide (ALSDES) at the N- and C-termini of EGFP. The PeV-A3 3C protease cleaves between the E and S of the ALSDES motif in the protein. After PeV-A3-EGFP is transcribed and translated in the infected cells, EGFP protein is cleaved from the PeV-A3-EGFP protein by the PeV-A3 3C protease. **c, d** The hPolI-PeV-A3-EGFP or mPolI-PeV-A3-EGFP plasmid was transfected into BHK-21 cells, and the culture supernatant prepared from the transfected cells was incubated with HuTu-80 cells. The expression of EGFP in these HuTu-80 cells was observed by fluorescence microscopy. Scale bars, 20 μm (**c**). The virus titers in the culture supernatant of HuTu-80 cells are shown as the mean log_10_ TCID_50_ (50% tissue culture infective dose) per 25 μl with s.d. from triplicate experiments. Data from one experiment representative of two independent experiments (*N* = 2) are shown. *P* value was calculated by an unpaired two-tailed *t*-test. ****P < 0.0001 (**d**). Source data are provided as a Source Data file.

HuTu-80 is a human cell line derived from duodenal adenocarcinoma and it is highly susceptible to PeV-A3 infection (Fig. 1a). To identify the PeV-A3 receptor in HuTu-80 cells, we performed genome-wide gene-knockout screening using the lentivirus CRISPR/Cas9 library. HuTu-80 cells were infected with the knockout library virus and then further infected with PeV-A3 (A308/99 strain). The most frequent knockout gene isolated from cells that survived PeV-A3 infection was human myeloid-associated differentiation marker (MYADM) (Supplementary Fig. 1a). MYADM is a 322-amino acid protein that contains 8 transmembrane domains and it is localized to the plasma membrane^11,12^ (Supplementary Fig. 1b). These features of MYADM are consistent with the requirements of a PeV-A3 receptor. Therefore, we further characterized MYADM as a candidate receptor for PeV-A3.

In contrast to HuTu-80 cells, BHK-21 cells derived from hamster kidney fibroblasts are not susceptible to infection with PeV-A3 (Fig. 1a). We hypothesized that the inability of PeV-A3 to infect BHK-21 cells may be due to the inability of PeV-A3 to enter BHK-21 cells. To investigate this hypothesis, we constructed a recombinant infectious PeV-A3 (PeV-A3-EGFP) that expresses enhanced green fluorescent protein (EGFP) when the PeV-A3-EGFP virus infects target cells. We constructed two PeV-A3-EGFP plasmids that express PeV-A3-EGFP under the control of the mouse RNA polymerase I promoter (mPolI) or the human RNA polymerase I promoter (hPolI) (Fig. 1b). It has been shown that the activation of transcription by the mPolI promoter in rodent cells is much higher than the activation of transcription by hPolI^13^. Thus, after transfection into BHK-21 cells, the mPolI-PeV-A3-EGFP plasmid is expected to produce more PeV-A3 mRNA and proteins in the transfected cells than the hPolI-PeV-A3-EGFP plasmid. These two PeV-A3-EGFP plasmids were transfected into BHK-21 cells, and the cells and culture supernatant were lysed over three freeze-thaw cycles. After centrifugation of the lysates, PeV-A3-susceptible human HuTu-80 cells were infected with supernatants containing PeV-A3-EGFP. The culture supernatant prepared from BHK-21 cells transfected with the mPolI-PeV-A3-EGFP plasmid, but not hPolI-PeV-A3-EGFP, produced infectious PeV-A3-EGFP in HuTu-80 cells, induced cytopathic effects (CPEs), and also induced the expression of EGFP (Fig. 1c, d). These results suggest that the inability of PeV-A3 to infect BHK-21 cells is most likely due to a defect in the entry step of PeV-A3.

**Fig. 2:**
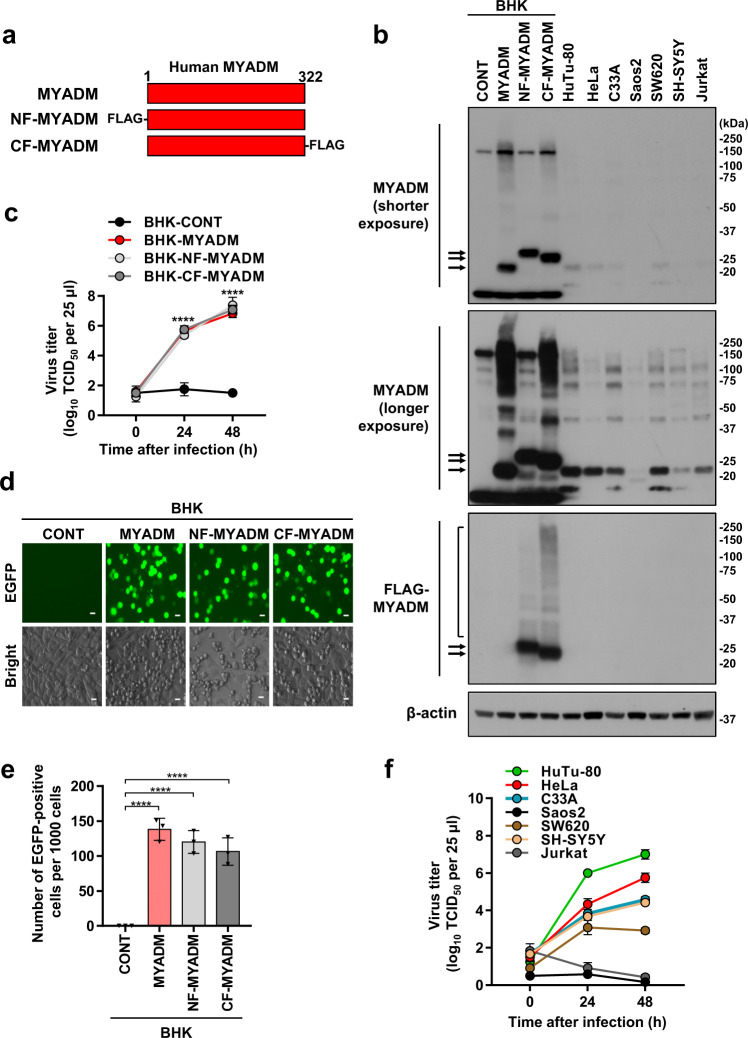
MYADM expression on PeV-A3 non-susceptible cells induces PeV-A3 infection. **a** A schematic illustration of the structure of the human MYADM protein that was used. **b** Cell lysate was prepared from the indicated cells, and the expression levels of endogenous MYADM, FLAG-MYADM, and β–actin in cell lysates were characterized by Western blotting. Representative Western blots of two independent experiments (*N* = 2) are shown; the two experiments gave similar results. **c**, **f** The indicated cells were infected with PeV-A3 (A308/99) at an MOI of 0.01, and the amount of virus (virus titer) in the culture supernatant was measured at 0, 24 and 48 h after infection. The virus titers in the culture supernatant of BHK-CONT (black circle), BHK-MYADM (red circle), BHK-NF-MYADM (light gray circle), BHK-CF-MYADM (dark gray circle), HuTu-80 (green circle), HeLa (red circle), C33A (blue circle), Saos2 (black circle), SW620 (brown circle), SH-SY5Y (peach circle) and Jurkat (dark gray circle) cells are shown as the mean ± s.d. from triplicate experiments. Data from one experiment representative of two independent experiments (*N* = 2) are shown. *P* values were calculated by a two-way ANOVA with Tukey’s multiple comparisons test. ****P < 0.0001 (**c**). **d**, **e** The indicated cells were infected with PeV-A3-EGFP. At 24 h after infection, the number of EGFP-positive cells per 1000 cells was determined by fluorescence microscopy and the ImageJ software program and is expressed as the mean ± s.d. from triplicate experiments. Data from one experiment representative of two independent experiments (*N* = 2) are shown. *P* values were calculated by a one-way ANOVA with Tukey’s multiple comparisons test. ****P < 0.0001 (**e**). Scale bars, 20 μm (**d**). Source data are provided as a Source Data file.

Based on these results, we investigated whether the expression of human MYADM on BHK-21 cells leads to PeV-A3 infection. To this end, we established BHK-21 cells that constitutively express human MYADM with FLAG-tag at the N-terminus (BHK-NF-MYADM) or C-terminus (BHK-CF-MYADM) or without FLAG-tag (BHK-MYADM), as well as vector control cells (BHK-CONT) by lentiviral transduction (Fig. 2a). A MYADM antibody detected MYADM proteins in three BHK-21 cells infected with the three MYADM lentiviruses, but not in BHK-CONT cells (Fig. 2b). The molecular weight of human MYADM in BHK-21 cells was somewhat smaller than expected (approximately 35 kDa). Using FLAG antibodies, MYADM of the same size was detected in BHK-21 cells expressing two FLAG-tagged MYADM, but not non-tagged MYADM (Fig. 2b). In addition, the FLAG antibody detected many high molecular weight MYADM proteins. Immunostaining of BHK-21 cells with FLAG antibodies revealed that MYADM localizes to the plasma membrane and cytoplasm (Supplementary Fig. 2). The previous studies also showed that MYADM localizes to the plasma membrane^11,12^.

After PeV-A3 infection, the three BHK-MYADM cells showed a productive PeV-A3 infection, whereas BHK-CONT cells did not (Fig. 2c). In addition, the PeV-A3 infection of BHK-MYADM cells, but not that of BHK-CONT cells, induced a significant increase in PeV-A3 RNA in the culture supernatant (Supplementary Fig. 3a). Crystal violet staining showed that a PeV-A3 infection for 48 h induced significant CPEs in BHK-MYADM cells, but not BHK-CONT cells (Supplementary Fig. 3b). Moreover, the infection of three BHK-MYADM cells with PeV-A3-EGFP virus, induced the expression of EGFP, but not in BHK-CONT cells (Fig. 2d, e; Supplementary Fig. 3c, d). These results indicate that the expression of human MYADM confers PeV-A3 infection to hamster BHK-21 cells, which are originally resistant to PeV-A3 infection.

Next, we examined the expression of MYADM in seven human cell lines derived from different origins, including HuTu-80 cells, to determine whether its expression in these cells is involved in their susceptibility to PeV-A3 infection (Fig. 2b, f). These cell lines were derived from duodenal adenocarcinoma (HuTu-80), cervical adenocarcinoma (HeLa), cervical carcinoma (C33A), osteosarcoma (Saos2), colon adenocarcinoma (SW620), neuroblastoma (SH-SY5Y) and acute T-cell leukemia (Jurkat). Six of the seven cell lines expressed MYADM protein, with a molecular weight similar to that of non-tagged human MYADM in BHK-21 cells (Fig. 2b). With the exception of Jurkat cells, these cell lines produced infectious PeV-A3. In contrast, Saos2 cells, in which MYADM was not detected, did not produce infectious PeV-A3. These results showed that the expression levels of MYADM in these seven cell lines, with the exception of Jurkat, correlated well with the extent of PeV-A3 infection in these cells. These results suggest that MYADM is required to induce susceptibility to PeV-A3 infection in these human cells. On the other hand, despite the expression of MYADM, Jurkat cells did not produce infectious PeV-A3. These results suggest that MYADM is necessary, but not sufficient to induce PeV-A3 infection susceptibility in human cells, and that a factor (or factors) other than MYADM is thus required for PeV-A3 infection.

### MYADM knockout abrogates PeV-A3 infection in PeV-A3-susceptible cells

**Fig. 3:**
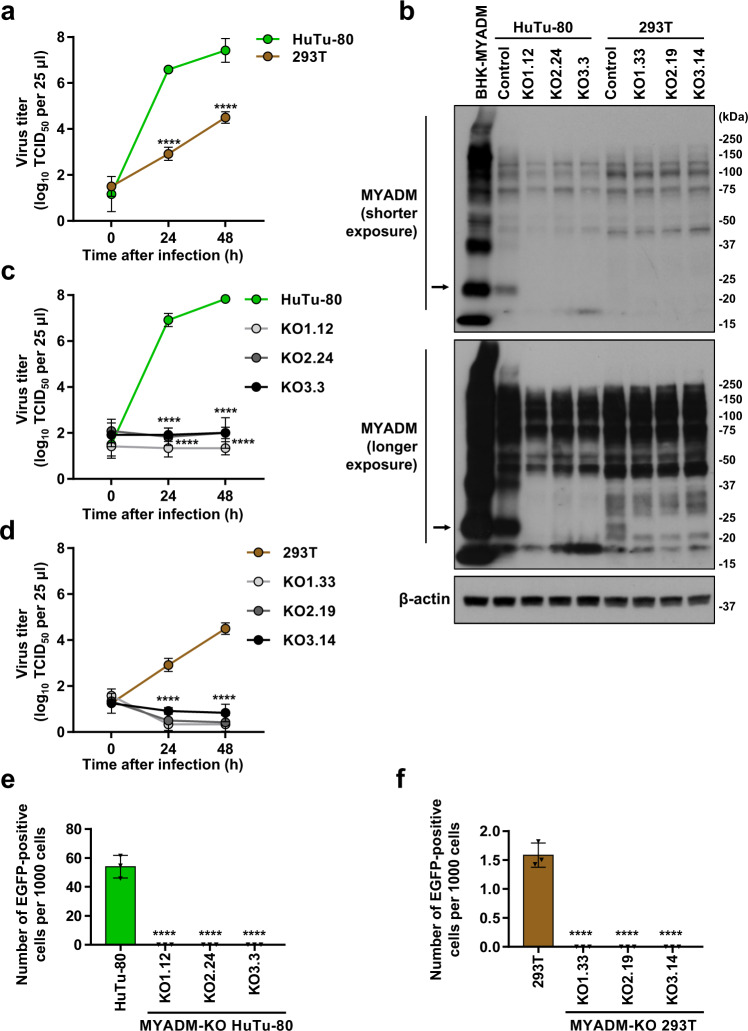
MYADM-KO abrogates PeV-A3 infection to susceptible cells. **a, c, d** The indicated cells were infected with PeV-A3 (A308/99) at an MOI of 0.01, and the amount of virus (virus titer) in the culture supernatant was measured at 0, 24 and 48 h after infection. The virus titers in the culture supernatant of HuTu-80 (green circle), 293T (brown circle), HuTu-80-MYADM-KO1.12 (light gray circle), KO2.24 (dark gray circle), KO3.3 (black circle), 293T-MYADM-KO1.33 (light gray circle), KO2.19 (dark gray circle), KO3.14 (black circle) cells are shown as the mean ± s.d. from triplicate experiments. Data from one experiment representative of two independent experiments (*N* = 2) are shown. *P* values were calculated by a two-way ANOVA with Sidak’s (**a**) or Tukey’s (**c, d**) multiple comparisons test. ****P < 0.0001. **b** Cell lysates were prepared from the indicated cells, and the amount of MYADM and β–actin in cell lysate was characterized by Western blotting. Arrows indicate endogenous MYADM in HuTu-80 and 293T, and exogenous MYADM in BHK-MYADM. Representative Western blots of two independent experiments (*N* = 2) are shown; the two experiments gave similar results. **e, f** The indicated cells were infected with PeV-A3-EGFP. At 24 h after infection, the number of EGFP-positive cells was assessed by fluorescence microscopy (Supplementary Fig. 4a, b) and the ImageJ software program, and the number of EGFP-positive cells per 1000 cells is shown as the mean ± s.d. from triplicate experiments. Data from one experiment representative of two independent experiments (*N* = 2) are shown. *P* values were calculated by a one-way ANOVA with Tukey’s multiple comparisons test. ****P < 0.0001. Source data are provided as a Source Data file.

Human 293T cells were susceptible to PeV-A3 infection, but it produced much less PeV-A3 in comparison to that observed in HuTu-80 cells (Fig. 3a). To clarify the role of MYADM in PeV-A3 infection of HuTu-80 and 293T cells, we knocked out the expression of MYADM in these two cell lines using CRISPR/Cas9 (MYADM-KO). Using a MYADM antibody, MYADM proteins were detected in HuTu-80 cells and 293T cells, but not in any of the three MYADM-KO cells, and the amount of MYADM in HuTu-80 cells was much higher than that in 293T cells (Fig. 3b). Productive PeV-A3 infection was observed in HuTu-80 and 293T cells, but not in any of the three MYADM-KO cells (Fig. 3c, d). In addition, MYADM-KO abrogated the PeV-A3-EGFP-induced EGFP expression in HuTu-80 and 293T cells (Fig. 3e, f; Supplementary Fig. 4a, b). Consistently, PeV-A3-induced CPEs were detected in HuTu-80 cells but not in MYADM-KO cells (Supplementary Fig. 4c). These results indicate that HuTu-80 and 293T cells produce high and low levels of PeV-A3, respectively, but both cell lines require MYADM for productive PeV-A3 infection.

It is known that virus strains cultured in vitro for long periods of time sometimes behave differently from viruses present in virus-infected individuals. Therefore, we investigated the role of MYADM in the infection of PeV-A3 (Niigata-423/13), which was isolated from PeV-A3-infected individuals and cultured for a short period of time (Supplementary Fig. 5). Like PeV-A3 (A308/99 strain), PeV-A3 (Niigata-423/13) induced productive PeV-A3 infection in HuTu-80 cells and BHK-21 cells expressing human MYADM, but not in MYADM-KO HuTu-80 or BHK-21 cells without FLAG-MYADM. These results indicate that MYADM is essential for the productive infection of a freshly isolated strain of PeV-A3 (Niigata-423/13).

### MYADM is an essential protein for the infection of six PeV-A genotypes

**Fig. 4:**
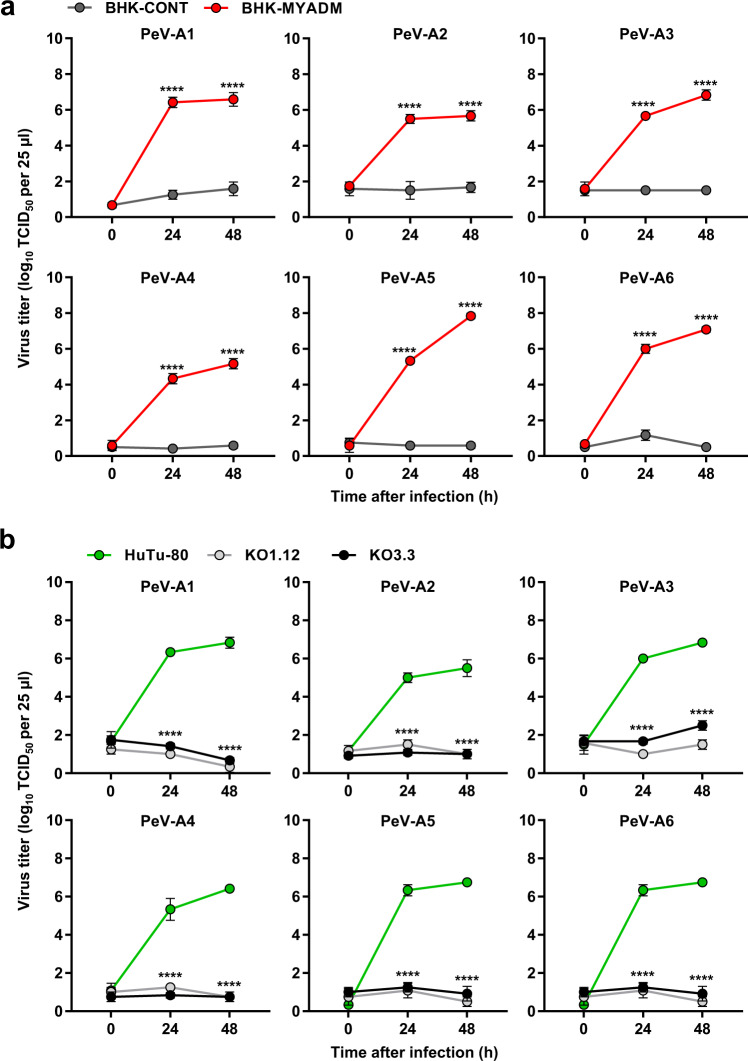
MYADM is essential for infection of six PeV-A genotypes. **a** BHK-MYADM (red circle) and BHK-CONT (dark gray circle) cells were infected with the indicated PeV-A genotypes at an MOI of 0.01, and the amount of virus (virus titer) in the culture supernatant was measured at 0, 24 and 48 h after infection. The virus titers in the culture supernatant are shown as the mean ± s.d. from triplicate experiments. Data from one experiment representative of two independent experiments (*N* = 2) are shown. **b** HuTu-80 (green circle), MYADM-KO1.12 (light gray circle) and KO3.3 (black circle) cells were infected with the indicated PeV-A genotypes at an MOI of 0.01, and the virus titer in the culture supernatant was measured at 0, 24 and 48 h after infection. The virus titers in the culture supernatant are shown as the mean ± s.d. from triplicate experiments. Data from one experiment representative of two independent experiments (*N* = 2) are shown. *P* values were calculated by a two-way ANOVA with Sidak’s multiple comparisons test in (**a, b**). ****P < 0.0001 (**a, b**). Source data are provided as a Source Data file.

There are 19 PeV-A genotypes, including PeV-A3. In the case of PeV-A1, integrins (α_v_β_1_, α_v_β_3_, and α_v_β_6_) have been reported to act a receptor for PeV-A1 infection^14–16^, and these integrins interact with the integrin binding motif of the arginine-glycine-aspartic acid (RGD) tripeptide in the C-terminal portion of VP1. In contrast, PeV-A3 does not have the RGD tripeptide motif in the VP1 protein^17,18^. To determine whether PeV-As other than PeV-A3 use MYADM for their infection, we selected five PeV-A genotypes (PeV-A1, 2, 4–6). Interestingly, as seen in PeV-A3, the five chosen PeV-As showed a productive infection of BHK-MYADM cells, but not BHK-CONT cells (Fig. 4a). Furthermore, in HuTu-80 cells, productive infection with these five PeV-As was abrogated by MYADM-KO (Fig. 4b). In addition, these five PeV-As induced CPEs in BHK-MYADM and HuTu-80 cells, but not in BHK-CONT or MYADM-KO HuTu-80 cells (Fig. 4b; Supplementary Fig. 6). These results indicate that MYADM is a cellular factor required for infection with the six genotypes of PeV-A, including PeV-A3.

### PeV-A3 VP0 capsid protein interacts with human MYADM

**Fig. 5:**
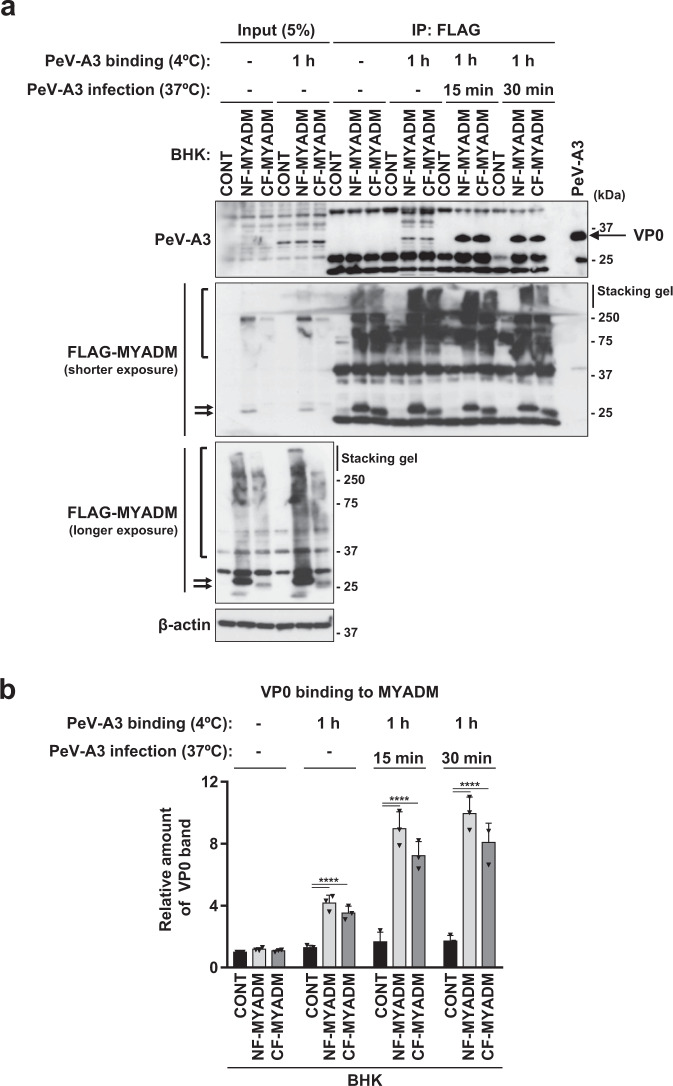
PeV-A3 capsid proteins interact with MYADM. **a**, **b** The indicated cells were incubated with concentrated PeV-A3 (A308/99) at 4 °C for 1 h, washed with PBS, and then incubated at 37 °C for 0, 15 or 30 min. The cells were then lysed with NP-40 lysis buffer and cell lysates were immunoprecipitated with anti-FLAG antibodies. Immunoprecipitates (IP) and pre-IP lysates (INPUT) were characterized by Western blotting with the indicated antibodies. The intensity of the band corresponding to VP0 protein on Western blotting was determined by densitometry, and is expressed as the mean ± s.d. from three independent experiments (*N* = 3). Data from one experiment representative of three independent experiments are shown. *P* values were calculated by a two-way ANOVA with Tukey’s multiple comparisons test in (**b**). ****P < 0.0001(**b**). Source data are provided as a Source Data file.

PeV-A infection is initiated by the interaction of viral capsid proteins with viral receptor proteins on the host cell membrane^10^. Therefore, we next investigated whether PeV-A3 proteins interact with MYADM. BHK-21 cells with FLAG-MYADM (NF-MYADM, CF-MYADM) or without FLAG-MYADM were treated with PeV-A3 prepared from virus-infected HuTu-80 cells at 4 °C for 1 h. After washing, the cells were further incubated at 37 °C for 15–30 min to induce virus entry and cell lysates were prepared from these cells. The FLAG-MYADM in cell lysates was then immunoprecipitated with anti-FLAG, and the immunoprecipitants were characterized by polyclonal anti-PeV-A3 serum. After treating the cells with PeV-A3 at 4 °C, anti-PeV-A3 serum detected a protein of approximately 35-kDa in FLAG-immunoprecipitate from BHK-21 cells with FLAG-MYADM, but not from BHK-21 cells without FLAG-MYADM (Fig. 5). The detection of the 35-kDa protein in FLAG-MYADM-expressing cells was increased by incubating these cells at 37 °C. We purified PeV-A3 by cesium chloride density-gradient centrifugation and separated PeV-A3 by polyacrylamide gel electrophoresis (PAGE). Mass spectrometry showed that the 35-kDa band in PAGE contained PeV-A3 VP0 protein (Supplementary Fig. 7). These results suggested that the PeV-A3 VP0 capsid protein interacts with MYADM on the plasma membrane of PeV-A3-susceptible cells. Interestingly, after BHK-21 cells were treated with PeV-A3 at 4 °C, VP0 proteins bound to BHK-21 cells even in the absence of the FLAG-MYADM protein (see the VP0 band of the input detected by the anti-PeV-A3 serum in Fig. 5a).

### One extracellular domain of human MYADM determines PeV-A3 infection to human cells

**Fig. 6:**
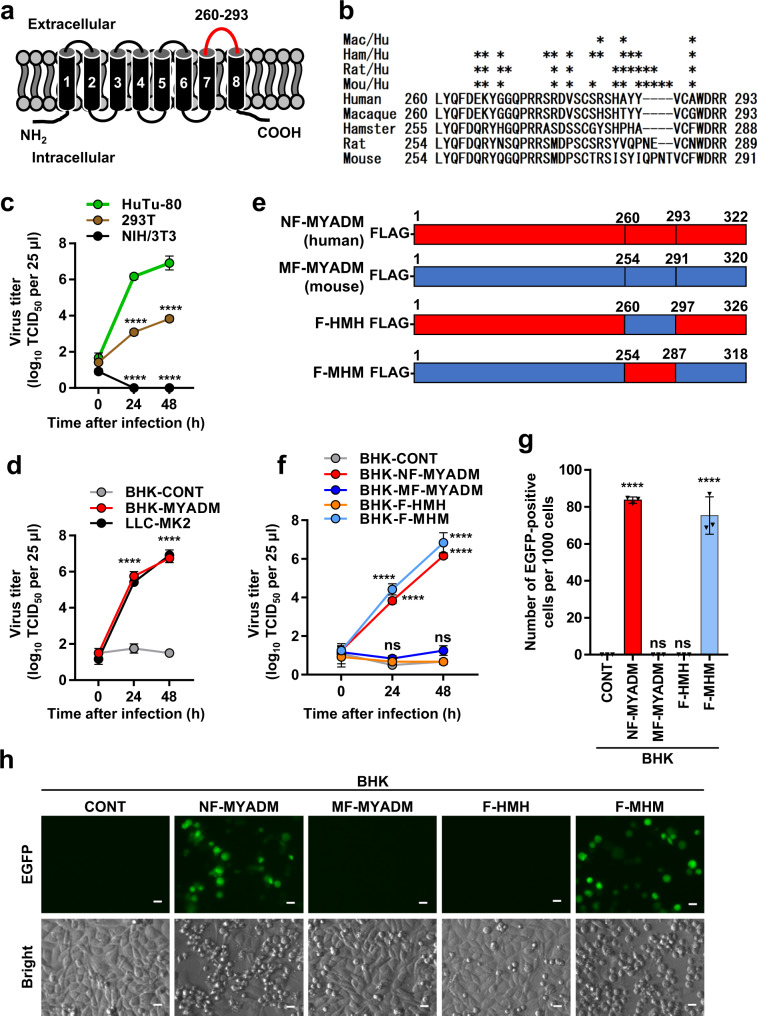
The fourth extracellular domain of human MYADM is essential for PeV-A3 infection. **a** A schematic illustration of the structure of human MYADM. **b** Amino acid sequences of the fourth extracellular domain of MYADM in five organisms. The accession numbers are as follows: human MYADM, AY037147; macaque MYADM, XM_015124836; hamster MYADM, XM_005084151; rat MYADM, AY344060; and mouse MYADM, AK089538. *Amino acid sequences that differ from human MYADM are marked with an asterisk. **c, d, f** Indicated cells were infected with PeV-A3 (A308/99) at an MOI of 0.01, and the amount of virus (virus titer) in the culture supernatant of HuTu-80 (green circle), 293T (brown circle), NIH/3T3 (black circle), BHK-CONT (light gray circle), BHK-MYADM (red circle), LLC-MK2 (black circle), BHK-NF-MYADM (red circle), BHK-MF-MYADM (blue circle), BHK-F-HMH (orange circle) and BHK-F-MHM (light blue circle) cells was measured at 0, 24 and 48 h after infection. The virus titers in the culture supernatant are shown as the mean ± s.d. from triplicate experiments. Data from one experiment representative of two independent experiments (*N* = 2) are shown. *P* values were calculated by a two-way ANOVA with Tukey’s multiple comparisons test. ****P < 0.0001. ns: not significant. **e** A schematic illustration of the structures of the chimeric MYADM proteins in humans and mice. **g, h** Indicated cells were infected with PeV-A3-EGFP. At 24 h after infection, the number of EGFP-positive cells per 1000 cells was determined by fluorescence microscopy and the ImageJ software program and is expressed as the mean ± s.d. from triplicate experiments. Data from one experiment representative of two independent experiments (*N* = 2) are shown. *P* values were calculated by a one-way ANOVA with Tukey’s multiple comparisons test. ****P < 0.0001. ns: not significant (**g**). Scale bars, 20 μm (**h**). Source data are provided as a Source Data file.

**Fig. 7:**
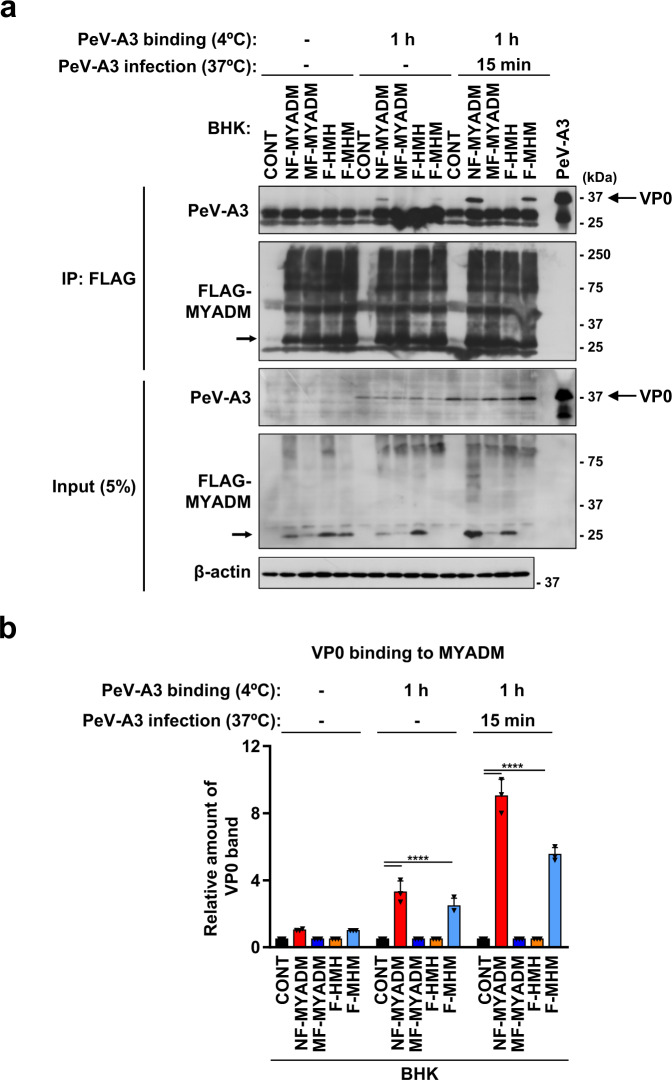
PeV-A3 capsid proteins interact with the fourth extracellular domain of human MYADM. **a, b** The indicated cells were incubated with concentrated PeV-A3 (A308/99) at 4 °C for 1 h, washed with PBS, and then incubated at 37 °C for 15 min. Cells were lysed with NP-40 lysis buffer and cell lysates were immunoprecipitated with anti-FLAG antibodies. Immunoprecipitates (IP) and pre-IP lysates (INPUT) were characterized by Western blotting with the indicated antibodies. The intensity of the band corresponding to VP0 protein on Western blotting was determined by densitometry, and is expressed as the mean ± s.d. from three independent experiments (*N* = 3). Data from one experiment representative of three independent experiments are shown. *P* values were calculated by a two-way ANOVA with Tukey’s multiple comparisons test in (**b**). ****P < 0.0001(**b**). Source data are provided as a Source Data file.

Human MYADM has four extracellular domains, of which the fourth domain has the longest amino acid sequence (Fig. 6a). PeV-A3 did not infect hamster BHK-21 cells (Figs. 1a and 2c), and the fourth extracellular domain of hamster MYADM differed from that of human MYADM by 12 amino acids (Fig. 6b). Based on these results, we speculated that the difference in the fourth extracellular domain of MYADM between humans and hamsters may determine an infection in human cells, but not in hamster BHK-21 cells with PeV-A3. Like hamster BHK-21, mouse NIH/3T3 cells were not sensitive to PeV-A3 infection (Fig. 6c). Like hamster MYADM, mouse MYADM differs from human MYADM by 10 amino acid substitutions and one insertion consisting of four amino acids. Since the cDNA of mouse MYADM was available, we constructed two chimeric constructs between human MYADM and mouse MYADM with FLAG-tag at the N-terminus (Fig. 6b, e). Four BHK-21 cell lines expressing human MYADM, mouse MYADM, and two chimeric MYADM proteins were established. The expression of MYADM protein was detected in BHK-21 cells transduced with any of the four MYADM lentiviruses, but not in the control BHK-21 (BHK-CONT) cells transduced with the empty vector virus (Fig. 7a). As expected, the incubation of BHK-21 expressing mouse MYADM (MF-MYADM) with PeV-A3 did not produce infectious PeV-A3 (Fig. 6f). However, BHK-21 expressing F-MHM, a mouse-based chimeric MYADM with the fourth extracellular domain of human MYADM, produced PeV-A3 to the same extent as human MYADM (Fig. 6f). In contrast, BHK-21 expressing F-HMH, a human-based chimeric MYADM with the fourth extracellular domain of mouse MYADM, did not produce PeV-A3 (Fig. 6f). Furthermore, infection with PeV-A3-EGFP induced the expression of EGFP and CPEs in BHK-21 cells expressing human MYADM (NF-MYADM) or F-MHM, but not mouse MYADM (MF-MYADM) or F-HMH (Fig. 6g, h). These results indicate that the differences in the fourth extracellular domain of MYADM between humans and mice determine the species-specific infection of PeV-A3.

Next, we investigated whether the fourth extracellular domain of human MYADM is involved in the binding to PeV-A3 VP0 protein. BHK-21 cells expressing the four FLAG-MYADM proteins used above were treated with PeV-A3, and the binding of FLAG-MYADM to VP0 was analyzed using anti-FLAG antibodies. Anti-FLAG antibodies immunoprecipitated comparable amounts of MYADM proteins from cells expressing the four MYADM proteins (Fig. 7). Human MYADM (NF-MYADM) bound to VP0 after the incubation of cells with PeV-A3, whereas mouse MYADM (MF-MYADM) and human-based chimeric MYADM (F-HMH) with the fourth extracellular domain of mouse MYADM, did not bind to VP0. On the other hand, mouse-based chimeric MYADM (F-MHM), which has the fourth extracellular domain of human MYADM, bound to VP0 when cells were incubated with PeV-A3 at 4 °C, and the amount of binding increased when incubated at 37 °C, although the amount of binding was slightly less than that of human MYADM. These results indicate that the fourth extracellular domain of human MYADM is essential for the productive infection of human cells with PeV-A3, by mediating the binding of human MYADM to the VP0 protein of PeV-A3, and that this domain determines the infection of human cells with PeV-A3, but not that of mouse or hamster cells.

PeV-A3 productively infected human and monkey cells (LLC-MK2), but not mouse or hamster cells (BHK-21) (Fig. 6c, d). Monkey MYADM differs from human MYADM by three amino acids in the fourth extracellular domain (Fig. 6b). Mouse MYADM differs from human MYADM by 10 amino acids and one insertion in the fourth extracellular domain. Hamster MYADM differs from human MYADM by 12 amino acids in the fourth extracellular domain. Taken together, these results suggest that the seven amino acids of the fourth extracellular domain of human and monkey MYADM, which differ from hamster and mouse MYADM, are likely to play a critical role in the binding of VP0 to MYADM and for PeV-A3 infection (Fig. 6b).

## Discussion

In this study, we found that MYADM is essential for productive PeV-A3 infection in human cells (Figs. 2 and 3). We also found that the VP0 capsid protein of PeV-A3 binds to MYADM and that this binding is closely associated with productive PeV-A3 infection (Figs. 5−7). These and other results suggest that MYADM is an essential host factor for PeV-A3 entry into human cells. Five human cell lines of different origins showed productive PeV-A3 infection; all of these cell lines expressed MYADM, while one cell line with no detectable MYADM expression showed no infection (Fig. 2), suggesting that the expression of MYADM determines PeV-A3 infection in cells of different origins (Figs. 2b, f and 3). A previous study showed that the MYADM mRNA expression varies between cell types^19^ and tissue types^20^. Taken together, the present results suggest that the differential expression of MYADM by cell type may play an important role in the tissue-specific infection of PeV-A3.

BHK-21 cells were resistant to PeV-A3 infection, but when transfected with a plasmid expressing PeV-A3 RNA and protein, infectious PeV-A3 was generated. These results indicate that BHK-21 cells are defective in the early stages of viral infection, such as virus attachment to the cells or viral capsid disassembly (virus uncoating) (Fig. 1). The VP0 capsid protein interacted weakly with the extracellular domain of MYADM on the plasma membrane at 4 °C, and such interaction was significantly augmented by incubation at 37 °C (Figs. 5 and 7). These results suggest that VP0 interacts with MYADM more strongly after PeV-A3 virion entry into host cells than during the initial attachment of PeV-A3 virions to the host cells. Taken together, these results suggest that MYADM is likely to be important in an uncoating stage of PeV-A3 infection.

The present study revealed that all six PeV-A genotypes examined, including PeV-A3, utilize MYADM as an essential host factor for PeV-A infection. PeV-A3 causes serious disease, such as meningoencephalitis, in neonates and young infants, which is rarely seen in other PeV-A genotypes^1^. Therefore, MYADM alone does not explain the high pathogenicity specific to PeV-A3. PeV-A1 uses integrins (α_v_β_1_, α_v_β_3_, and α_v_β_6_) as attachment receptors during PeV-A1 infection^14–16^, by the binding of these integrins with RGD motif in the PeV-A1 VP1 protein. In addition, PeV-A2, PeV-A4, PeV-A5, and PeV-A6 also have an RGD motif in their respective VP1. In contrast, the VP1 of PeV-A3 does not have an RGD motif^17,18^. Therefore, PeV-A3 may use a different attachment receptor from PeV-As with an RGD motif, and this differential use of attachment receptors may be involved in the difference in the pathogenicity of PeV-As.

MYADM has been shown to play a role in immune and inflammatory responses. For instance, in endothelial cells, MYADM controls cell membrane permeability and the inflammatory response by regulating ICAM-1 (adhesion molecule)-dependent cell to cell interactions^21^. In addition, in the HeLa epithelial cell line, MYADM controls cell spreading and migration by controlling the activity of the small GTPase Rac1^12^. These results suggest that the PeV-A3 infection of cells through MYADM may alter the function of MYADM, thereby modulating both immune and inflammatory responses. Further studies are needed to clarify the involvement of MYADM in the immune response to PeV-As and pathogenicity.

## Methods

### Cell culture

Human HuTu-80 cells (HTB-40) were derived from human duodenal adenocarcinoma, SW620 (CCL-227) cells were derived from human colon adenocarcinoma and were obtained from American Type Culture Collection. 293T^22^ cells were originally derived from human embryonic kidney tissue. HeLa^23^ cells were derived from human cervical adenocarcinoma. C33A^22^ cells were derived from human cervical carcinoma. Saos2^22^ cells were derived from human osteosarcoma. SH-SY5Y^24^ cells were derived from human neuroblastoma. Jurkat^22^ cells were derived from human acute T-cell leukemia. Syrian hamster BHK-21^25^ cells were derived from a baby hamster kidney. LLC-MK2^26^ cells were derived from a rhesus monkey kidney. NIH/3T3^27^ cells were derived from a mouse embryo. HuTu-80, BHK-21 and LLC-MK2 cells were cultured in Eagle’s minimal essential medium (M4655, Sigma) supplemented with 10% fetal calf serum (FCS) (10270106, Gibco), penicillin-streptomycin-glutamine (PSG) (10378016, Gibco), and MEM non-essential amino acids (NEAA) (11140050, Gibco) (10% FCS-EMEM). 293T, HeLa, C33A, Saos2 and SW620 cells were cultured in Dulbecco′s modified Eagle medium (DMEM) (11965092, Gibco) supplemented with 10% FCS, PSG, and NEAA (10% FCS-DMEM). SH-SY5Y cells were cultured in equal amounts of DMEM and Opti-MEM (31985070, Gibco) supplemented with 5% FCS, PSG, and NEAA (5% FCS-DMEM-Opti-MEM). Jurkat cells were cultured in RPMI-1640 medium (R8758, Sigma) supplemented with 10% FCS, PSG, and 2-mercaptoethanol (2-ME) (21985023, Gibco) (10% FCS-RPMI). NIH/3T3 cells were cultured in DMEM supplemented with 10% calf serum (16170078, Gibco), PSG, and NEAA (10% CS-DMEM).

### Viruses and the virus titer

PeV-A1 (Harris strain)^28^, PeV-A2 (Williamson strain)^28^, PeV-A3 (A308/99 strain)^17^, PeV-A4 (K251176-02 strain)^29^, PeV-A5 (CT86-6760 strain)^30^ and PeV-A6 (NII561-2000 strain)^26^ were provided by H. Shimizu, National Institute of Infectious Diseases. PeV-A3 strains (Niigata-422/13 and Niigata-423/13) were isolated from febrile infants. To prepare appropriate stock viruses for the infection assays, LLC-MK2 cells were infected with the virus for two days, and the culture supernatant was collected. Cell debris in the supernatant was removed by centrifugation. The virus titer (50% Tissue Culture Infectious Dose [TCID_50_]) in the supernatant was measured using LLC-MK2 cells and it was determined using the Reed−Muench method^31^.

### Quantification of virus infection

Cells were cultured in 6-well plates for three days. Cells at 90–100% confluence were infected with PeV-A with a multiplicity of infection (MOI) of 0.01 at 37 °C for 1 h. The cells were then washed 3 times with phosphate-buffered saline (PBS) and further cultured for 0–48 h in the medium described below for each cell. The media used for cell culture after virus infection were 2% FCS-EMEM for MYADM-KO, wild-type HuTu-80, BHK-21 and LLC-MK2, 2% FCS-DMEM for MYADM-KO, wild-type 293T, HeLa, C33A, Saos2 and SW620 cells, 2% FCS-EMEM with 800 μg/ml G418 (10131035, Gibco or 09380, Nacalai Tesque) for BHK-21 cells expressing various MYADM proteins, and BHK-CONT, 5% FCS-DMEM-Opti-MEM for SH-SY5Y, 10% FCS-RPMI for Jurkat, and 10% CS-DMEM for NIH/3T3 cells. The culture supernatant was collected from virus-infected cells, and the cell debris in the supernatant was removed by centrifugation. The virus titer in the supernatant was measured using a TCID_50_ assay, as described above. The assay was performed in triplicate, and the data are expressed as the mean ± standard deviation (s.d.).

### Genome-wide gene-knockout screening using the lentivirus CRISPR/Cas9 library

A commercial CRISPR gene-knockout lentivirus library (73179, Addgene) for the human genome was used. The library targets 19,114 human genes, with four different target sequences per gene. HuTu-80 cells were infected with gene-knockout library lentiviruses at an MOI of 1. Four days later, the cells were further infected three times with PeV-A3 (A308/99) at an MOI of 0.1 for 4–8 days. Genomic DNA was extracted from pooled surviving cells using the Kaneka Easy DNA Extraction Kit version 2 (KN-T110005, Kaneka). From this genomic DNA, a 160-bp DNA fragment corresponding to the gRNA regions in surviving cells was amplified by polymerase chain reaction (PCR) using the forward primer (P5) 5′-TTGTGGAAAGGACGAAACACCG-3′ and the reverse primer (P7) 5′-CCAATTCCCACTCCTTTCAAGACCT-3′. Pooled PCR products of 160 bp in size were sequenced by the TruSeq Nano DNA preparation protocol with a NovaSeq 6000 sequencer. Adapter sequences and low-quality sequences in the read sequences were removed and the sequences containing the gRNA sequences corresponding to human genes in the Addgene list were counted (Macrogen).

### Plasmids

The pE3SP plasmid contains the murine RNA polymerase I promoter (mPolI) and murine terminator sequence (mTer)^32^ and was a gift from Dr. Beat Lutz of the University Medical Center Mainz, Mainz, Germany. The human RNA polymerase I promoter (hpolI) was amplified by PCR from genomic DNA of Jurkat cells (a human T-cell line). The mPolI promoter and its enhancer sequence in pE3SP were replaced with the hPolI promoter sequence by the DNA assembly method (Gibson Assembly Master Mix; E2611, New England Biolabs). Human MYADM cDNA was amplified by PCR from a cDNA library generated from HeLa cells using the In-Fusion SMARTer directional cDNA library construction kit (634933, Clontech Takara) and transferred to pENTR/D-TOPO (K240020, Thermo Fisher Scientific). Mouse MYADM cDNA was purchased from Horizon and transferred to pENTR plasmid by PCR and the In-Fusion method. Chimeric constructs between human and mouse MYADM were constructed by PCR and the In-Fusion method. CSII-EF-IN is a bicistronic lentiviral expression vector expressing a neomycin resistance gene. CSII-EF-IN was constructed by inserting the internal ribosome entry site (IRES) gene and the neomycin resistance gene from pQCXIN (631514, Clontech Takara) into CSII-EF-MCS. CSII-EF-IN was converted to a Gateway destination vector by the insertion of the RfA cassette (11828029, Thermo Fisher Scientific). MYADM cDNAs were transferred from pENTR to CSII-EF-IN using LR Clonase (11791020, Thermo Fisher Scientific). pBluescript II SK (+) was used as the cloning vector (212205, Agilent). CSII-EF-MCS was provided by Dr. H. Miyoshi of the RIKEN Tsukuba Institute. pCAG-HIVgp and pCMV-VSV-G-RSV-Rev were the lentiviral packaging plasmids and were also provided by Dr. H. Miyoshi.

### Construction of the infectious PeV-A3-EGFP production plasmid

First, we constructed a PeV-A3 producing plasmid. LLC-MK2 cells were infected with the PeV-A3 (A308/99), and PeV-A3 RNA was prepared from the culture supernatant of these LLC-MK2 cells using the QIAamp Viral RNA Mini Kit (52904, Qiagen). From this RNA the full-length PeV-A3 cDNA was amplified by reverse transcription PCR (RT–PCR) (PrimeScript II High Fidelity One Step RT-PCR Kit; R026, Takara Bio) and cloned into the hPolI expression vector (pE3SP) by the Gibson assembly method, and the resulting plasmid was designated hPolI-PeV-A3. The hPolI expression vector (pE3SP) contains hPolI DNA and mouse ribosomal gene transcription terminator (mTer) DNA. A DNA fragment containing hPolI, PeV-A3 cDNA and mTer in the hPolI-PeV-A3 plasmid was cloned into the pBluescript plasmid. The resulting plasmid pBS-PeV-A3 contained the hPolI promoter, the full-length PeV-A3 cDNA and mTer (Fig. 1b).

We next constructed a PeV-A3-EGFP expression plasmid (hPolI-PeV-A3-EGFP) that contained EGFP cDNA between PeV-A3 VP1 and 2A DNA. After PeV-A3-EGFP infects a cell, the 3C protease of PeV-A3 excises the EGFP protein from the PeV-A3-EGFP fusion protein. An EGFP DNA fragment with the PeV-A3 3C protease recognition sequence (-ALSDE/S-) at the N- and C-terminus was constructed by PCR and inserted between the PeV-A3 VP1 and 2A DNA of pBS-PeV-A3 via the Gibson assembly method. After confirming the DNA sequence, the resulting construct was designated hPolI-PeV-A3-EGFP. To produce the PeV-A3-EGFP virus, the hPolI-PeV-A3-EGFP plasmid was transfected into 293T cells using FuGENE 6 (E2691, Promega). Three days after transfection, the cells were lysed over three freeze-thaw cycles. After centrifugation virus-containing cell lysates were collected. These viruses were used to infect LLC-MK2 cells. Ten days after infection, the viruses in LLC-MK2 cells were harvested as described above and stored at −80 °C in a freezer before use in the infection assay.

To investigate the virus-producing activity of PeV-A3 RNA in hamster BHK-21 cells, the hPolI promoter of the hPolI-PeV-A3-EGFP plasmid was re-replaced with the mPolI promoter by the Gibson assembly method, and the resulting plasmid was designated mPolI-PeV-A3-EGFP. mPolI-PeV-A3-EGFP and hPolI-PeV-A3-EGFP plasmids were transfected into BHK-21 cells using Lipofectamine 2000 (11668019, Invitrogen). Seven days after transfection, the cells were lysed over three freeze-thaw cycles. After centrifugation, virus-containing cell lysates were collected. HuTu-80 cells prepared in 24-well plates were infected with these viruses to investigate the production of PeV-A3-EGFP.

### Quantification of PeV-A3-EGFP infection by fluorescence microscopy

Cells were cultured in 6-well plates for three days. Next, cells at 90–100% confluence were infected with PeV-A3-EGFP at an MOI of 0.1 at 37 °C for 1 h. The cells were then further cultured in low-serum medium for 24 h. As the low-serum medium, 2% FCS-EMEM was used for MYADM-KO and wild-type HuTu-80 cells, 2% FCS-DMEM was used for MYADM-KO and wild-type 293T cells, and 2% FCS-EMEM with 800 μg/ml G418 was used for BHK-21 cells expressing various MYADM proteins, and BHK-CONT cells. Images were captured using a BZ-X810 fluorescence microscope (Keyence) with one field captured per well. Image quantification was performed using the ImageJ software program (version 1.53e).

### Immunofluorescence staining

Cells were plated on glass coverslips in a 6-well plate, fixed with 4% paraformaldehyde in PBS at room temperature for 20 min, and then permeabilized with 0.1% Triton X-100 in PBS at room temperature for 5 min. Fixed cells were incubated with mouse anti-FLAG antibodies (1:200; F1804, Merck) at room temperature for 60 min, washed with PBS, and then incubated with the secondary anti-mouse immunoglobulin labeled with Alexa488 (1:500; A-11029, Thermo Fisher Scientific) and Hoechst 33258 (H3569, Thermo Fisher Scientific) for nuclear staining at room temperature for 60 min. Cell staining was evaluated using a BZ-X810 fluorescence microscope (Keyence) and a fluorescence analysis software program (BZ-II analyzer; Keyence).

### The establishment of BHK-21 cells expressing various MYADM proteins

293T cells were transfected with MYADM lentiviral vector and lentivirus packaging plasmids (pCAG-HIVgp, pCMV-VSV-G-RSV-Rev) by PEI Max plasmid transfection reagent (24765, Polysciences) and cultured for 72 h. The culture supernatant containing the lentiviruses was added to BHK-21 cells and cultured in selection medium containing 800 μg/ml G418.

### The establishment of MYADM-KO HuTu-80 and 293T cells

To generate CRISPR/Cas9 vectors for MYADM-KO cells, three gRNA sequences were selected from MYADM exon3 (5′-CAGGCCCGAAGATGACGTCGTGG-3′, 5′-CCTGTCCCACGGCCGTTCGCGGG-3′, and 5′-CCGTTCGCGGGACCACGCCATCG-3′) and cloned into the pX330-U6-Chimeric_BB-CBh-hSpCas9 plasmid (42230, Addgene)^33^. HuTu-80 or 293T cells were co-transfected with the CRISPR/Cas9 vector and pBS-Puro^34^ plasmid with FuGENE HD (E2311, Promega). Twenty-four hours after transfection, the cells were cultured in puromycin-containing media for 72 h and then replaced with puromycin-free media. MYADM-KO cells were cloned by limiting dilution and then were verified by Western blotting and genomic sequencing.

### Measurement of PeV-A3 RNA by quantitative RT-PCR (qRT-PCR)

Cells were infected with PeV-A3 (A308/99), as described above. PeV-A3 RNA was extracted from the culture supernatant of PeV-A3-infected cells with a QIAamp Viral RNA Mini Kit (52904, Qiagen) according to the manufacturer′s instructions. The PeV-A3 DNA fragment (PeV-A3 VP1 region) was amplified from PeV-A3 RNA by RT-PCR using the One Step TB Green PrimeScript RT-PCR Kit II (RR086, Takara Bio), and the amount of PeV-A3 DNA was quantified by comparison with a standard curve generated from PCR products of pBS-PeV-A3 plasmid (10^2^–10^6^ copies per μl), and the amount of PeV-A3 RNA in a sample was presented as log_10_ copies per μl. The Thermal Cycler Dice Real Time System (Takara Bio) was used for qRT-PCR. PCR was performed at 42 °C for 5 min, 95 °C for 10 s, and 40 cycles of 95 °C for 5 s and 60 °C for 30 s. The nucleotide sequence of the VP1 (pBS-PeV-A3 plasmid) forward primer was 5′-GACAACATCTTTGGTAGAGCTTGGT-3′, and that of the VP1 reverse primer was 5′-TTCTGCCTCCAGGTATCTCCAC-3′. The assay was performed in triplicate and all data are presented as the mean ± s.d.

### Quantification of virus-induced CPEs

Cells were cultured in 48-well plates and infected with PeV-A at an MOI of 0.1 at 37 °C for 1 h. After removal of the culture supernatant, virus-infected cells were further cultured in low-serum medium for two days. Cells adhering to the culture plate were fixed with 10% formaldehyde in PBS, and the amount of virus-induced CPE was measured by staining the cells with crystal violet (0.5% crystal violet, 25% methanol in H_2_O). As the low-serum medium, 2% FCS-EMEM was used for MYADM-KO and wild-type HuTu-80 cells, and 2% FCS-EMEM with 800 μg/ml G418 was used for BHK-MYADM and BHK-CONT cells.

### Western blotting

Cells were lysed in RIPA buffer (50 mM Tris-HCl, pH 8.0, 150 mM NaCl, 0.1% SDS, 1% Nonidet P-40, 1% sodium deoxycholate, Halt protease inhibitor cocktail (87786, Thermo Fisher Scientific)) and 2× SDS sample buffer (125 mM Tris-HCl, pH 6.8, 4% SDS, 10% sucrose, 0.01% bromophenol blue, 10% 2-ME), and the cell lysates were incubated at 4 °C overnight. Cell lysates were sonicated, centrifuged at 10,000 × *g* for 5 min at 4 °C, and the supernatant (20 μg) was separated by SDS-PAGE and electrophoretically transferred onto a PVDF membrane (Immobilon; 162-0177, Bio-Rad). PVDF membranes were incubated for 1 h in 5% skim milk in Tris-buffered saline (TBST; 20 mM Tris-HCl, pH 7.4, 150 mM NaCl, 0.2% tween-20) and further treated with the indicated antibodies diluted in Can Get dilution buffer (NKB-101, Toyobo). Immunoreactive bands in the PVDF membranes were detected using an enhanced chemiluminescence (ECL) detection system (ECL Western Blotting Detection Reagents; RPN2209, GE Healthcare, Pierce ECL Plus Western Blotting Substrate; 32132, Thermo Fisher Scientific) and visualized on Amersham Hyperfilm ECL (28906835, GE Healthcare). The following antibodies were used for immunostaining: rabbit polyclonal anti-MYADM (1:2000; NBP2-24494, Novus), mouse anti-FLAG (1:10,000; F1804, Merck), mouse monoclonal anti-β-actin (1:1000; sc-47778, Santa Cruz), anti-rabbit IgG conjugated to horse radish peroxidase (HRP) (1:20,000; 7074, Cell Signaling), and anti-mouse IgG conjugated with HRP (1:20,000; 170-6516, Bio-Rad).

### Preparation of polyclonal anti-PeV-A3 serum

Antiserum against PeV-A3 was prepared by Dr. Makoto Yamazaki (Denka Co. Ltd, Niigata, Japan). Briefly, LLC-MK2 cells were infected with PeV-A3 (Niigata-422/13), and the infected cells were cultured for five days. PeV-A3 in the culture supernatant was purified by cesium chloride (CsCl) density-gradient centrifugation^26^. The purified viruses were emulsified in equal amounts of Freund’s complete adjuvant and injected subcutaneously into the limbs of 1 rabbit 3 times (3, 5, 10 μg) at 2-week intervals. One and two weeks later, the purified virus (20, 40 μg) without adjuvant was again injected into the ear veins of an immunized rabbit. Seven days later, whole blood was collected from the immunized rabbit.

### Virus binding assay

HuTu-80 cells were infected with PeV-A3 (A308/99), and then cultured for two days. Viruses in the culture supernatant of virus-infected cells were concentrated by a centrifugal ultrafiltration filter using Amicon Ultra-15 (100 K) (UFC910024, Millipore). BHK-21 cells were cultured in 6-cm dishes (Corning), and cells at 90–100% confluence were infected with 500 µl of concentrated PeV-A3 at an MOI of 1 on ice for 1 h, washed with PBS, followed by additional incubation at 37 °C for 0–30 min. After washing 3 times with PBS, cells were lysed with ice-cold NP-40 lysis buffer (1% Nonidet P-40, 25 mM Tris-HCl, pH 7.2, 150 mM NaCl, 1 mM EDTA, 1 mM phenylmethylsulfonyl fluoride, 20 μg/ml aprotinin). The cell lysates were then centrifuged, and the soluble fractions of the cell lysates were immunoprecipitated with the anti-FLAG primary antibody (4 µg), and the immune complexes were precipitated with 20 µl of Protein G Sepharose (17061801, GE Healthcare). The beads were washed with NP-40 lysis buffer and boiled in 2× SDS sample buffer. After centrifugation, supernatants containing proteins released from the beads were separated by SDS-PAGE, transferred to a membrane, followed by blocking and incubation with antibodies as described in the Western blotting section. Polyclonal anti-PeV-A3 serum (1:1000; anti-Niigata-422/13 serum was a gift from M. Yamazaki) was used to detect PeV-A3 proteins.

### Identification of viral protein by mass-spectrometry

PeV-A3 (Niigata-422/13) viruses were purified by the above-described CsCl density-gradient centrifugation method^26^ and separated by 10–15% SDS-PAGE and the SDS-PAGE gel was stained with Coomassie Brilliant Blue (CBB). The gel containing a stained band around 35-kDa was cut out and the gel was washed 4 times with MilliQ water and 3 times with 50 mM ammonium bicarbonate (ABC) containing 50% acetonitrile and then dehydrated with 100% acetonitrile for 10 min. Proteins in gels were reduced and alkylated by incubation with 10 mM dithiothreitol in 50 mM ABC for 1 h at 56 °C and with 50 mM iodoacetamide in 50 mM ABC in a dark at room temperature for 45 min before being washed twice times with 50 mM ABC and incubated overnight with 90 µl of trypsin in 25 mM ABC for protein digestion.

The digested proteins were analyzed by a TripleTOF5600+ mass spectrometer (Sciex). Peak lists were generated by an AB SCIEX MS Data Converter and compared with the PeV-A3 database using the Mascot software program (version 2.6.0; Matrix Science). Trypsin was selected as the enzyme used, and the maximum number of missed cleavages allowed was set at 1. Carbamidomethylation on cysteine was selected as the fixed modification. Oxidized methionine and pyroglutamination of N-terminal Gln were searched for as variable modifications. The precursor mass tolerance was 20 ppm, and the ion tolerance of tandem mass spectrometry was 0.1 Da.

### Staining of SDS-PAGE gels with CBB

After SDS-PAGE, the gels were incubated in CBB solution (0.125% Coomasie blue, 50% methanol, 10% acetic acid, 60% distilled H_2_O) for 1 h, then incubated in CBB destaining buffer (10% acetic acid, 50% methanol, 40% distilled H_2_O) for 3–4 h until staining bands became visible.

### Statistical analyses

Statistical analyses were performed using the unpaired Student’s *t*-test (two-tailed), a two-way analysis of variance followed by Sidak’s multiple comparisons test, or a one-way or two-way analysis of variance followed by Tukey’s multiple comparisons test using the Prism7 software program (GraphPad Software). Data are presented as the mean and s.d.

### Reporting summary

Further information on research design is available in the Nature Portfolio Reporting Summary linked to this article.

## Supplementary information

Supplementary Information

Reporting Summary

## Data Availability

The DNA sequences of PeV-A3-EGFP generated in this study has been deposited in the DDBJ database under accession number LC723624. Source data are provided with this paper.

## Source data

Source Data
