# Supplementary material for: Myeloid-associated differentiation marker is an essential host factor for human parechovirus PeV-A3 entry
Source: Nat Commun. 2023 Mar 31;14:1817. doi: 10.1038/s41467-023-37399-8 (PMC10066301; doi:10.1038/s41467-023-37399-8)
Supplement: Supplementary file 1 — Supplementary Information [file 41467_2023_37399_MOESM1_ESM.pdf]

Supplementary Information

Myeloyed-associated differentiation marker is an essential host factor for human parechovirus PeV-A3 entry

Kanako Watanabe[1,9], Tomoichiro Oka[2,9], Hirotaka Takagi[3,9], Sergei Anisimov[4], Shun-ichi Yamashita[5], Yoshinori Katsuragi[6], Masahiko Takahashi[4], Masaya Higuchi[7], Tomotake Kanki[5], Akihiko Saitoh[8] and Masahiro Fujii[4] ✉

[1]Division of Laboratory Science, Niigata University Graduate School of Health Sciences, Niigata, Japan.

[2]Department of Virology II, National Institute of Infectious Diseases, Tokyo, Japan.

[3]Management Department of Biosafety, Laboratory Animal, and Pathogen Bank, National Institute of Infectious Diseases, Tokyo, Japan.

[4]Division of Virology, Niigata University Graduate School of Medical and Dental Sciences, Niigata, Japan.

[5]Department of Cellular Physiology, Niigata University Graduate School of Medical and Dental Sciences, Niigata, Japan.

[6]Faculty of Nursing, Niigata College of Nursing, Niigata, Japan.

[7]Department of Microbiology, Kanazawa Medical University School of Medicine, Ishikawa, Japan.

[8]Department of Pediatrics, Niigata University Graduate School of Medical and Dental Sciences, Niigata, Japan.

[9]These authors contributed equally to the study: Kanako Watanabe, Tomoichiro Oka, Hirotaka Takagi.

✉ email: fujiimas@med.niigata-u.ac.jp

a

	Target Gene ID	Target Gene Symbol	sgRNA Target Sequence	Mapped reads
1	91663	MYADM*	CTCCACGATGAGGATGATCA	1000355
2	91663	MYADM*	GCTATATGGCCACCGTACCC	813436
3	390265	OR10G7	GGAGATAGTCCTGCCGCTTG	329650
4	23308	ICOSLG	CATCCATAAACGGCTACCCC	312715
5	29843	SENP1	ATGACAAAATACGAACCTTG	205145
6	80011	FAM192A	CAGGAGCTGTGAAGCATAAG	173259
7	28978	TMEM14A	AAAAGACCAGCAATCAAAGA	171532
8	440730	TRIM67	CAGGCAGTACTCCATCAGTC	167123
9	339302	CPLX4	AGCTCAAGGGATGACTAGAG	153577
10	26526	TSPAN16	CAGACGACTATTCTACACAG	140914
11	84289	ING5	CTGGACCAGAGGACGGAAGG	119663
12	644538	SMIM10	TGCGGCTGTCGCGCCCGCAG	108880
13	218	ALDH3A1	TTCTCCACAGGAGTTCTACG	96657
14	10936	GPR75	CCAGCAATCAGACTGGACAT	96235
15	100137047	JMJD7	AGAGCGTTGCGGATAATGCA	92339
16	646174	C16orf90	CACCCCCCAACATCTACGAG	89303
17	64861	SNRK	TGTCTTTACGGGTGAAAAGG	84975
18	79717	PPCS	CTTCCTAGCCGCCGGCTACG	77177
19	140947	DCANP1	TGGAGTTGCAGAGTCCCCTG	70459
20	50700	RDH8	CCTCAAACTCGGTGACCACG	68062
21	387266	KRTAP5-3	GGGGTCTGTGGATTTCGTGG	66752
22	729420	LMO7DN	TGATTTCGCTGGAAGTGCGG	65248
23	91663	MYADM*	AGGAACTGGACATAGGTGGT	63487
		Non-Targeting Control	ATAGCTAAAGTTGATGTGTA	62839

b

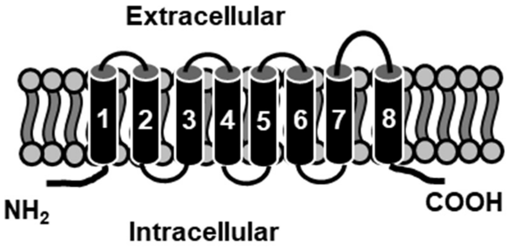

Supplementary Fig. 1 MYADM was identified as a candidate host factor for PeV-A3 entry by a Genome-wide gene knockout screen. a Knockout genes enriched in surviving cells after PeV-A3 (A308/99) infection. *Three MYADM gRNAs were detected in knockout genes enriched in surviving cells after PeV-A3 (A308/99) infection. **b** A schematic illustration of the structure of human MYADM.

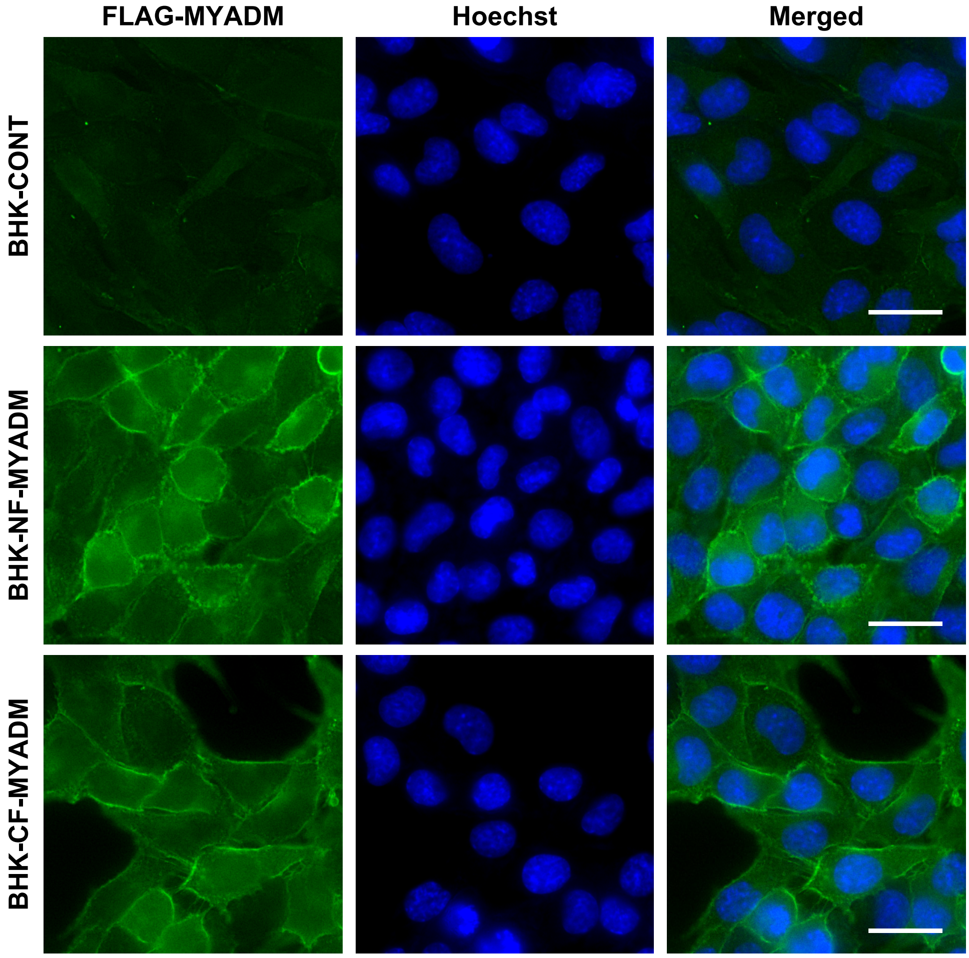

Supplementary Fig. 2 Immunofluorescence staining of BHK-21 cells expressing human FLAG-tagged MYADM. The indicated BHK-21 cells were stained with an anti-FLAG antibody (green) and Hoechst (blue). Cell staining was determined by fluorescence microscopy with an oil immersion lens. Scale bars, 25 μm. Representative micrographs of three independent experiments ($N = 3$) are shown; the three experiments gave similar results. Source data are provided as a Source Data file.

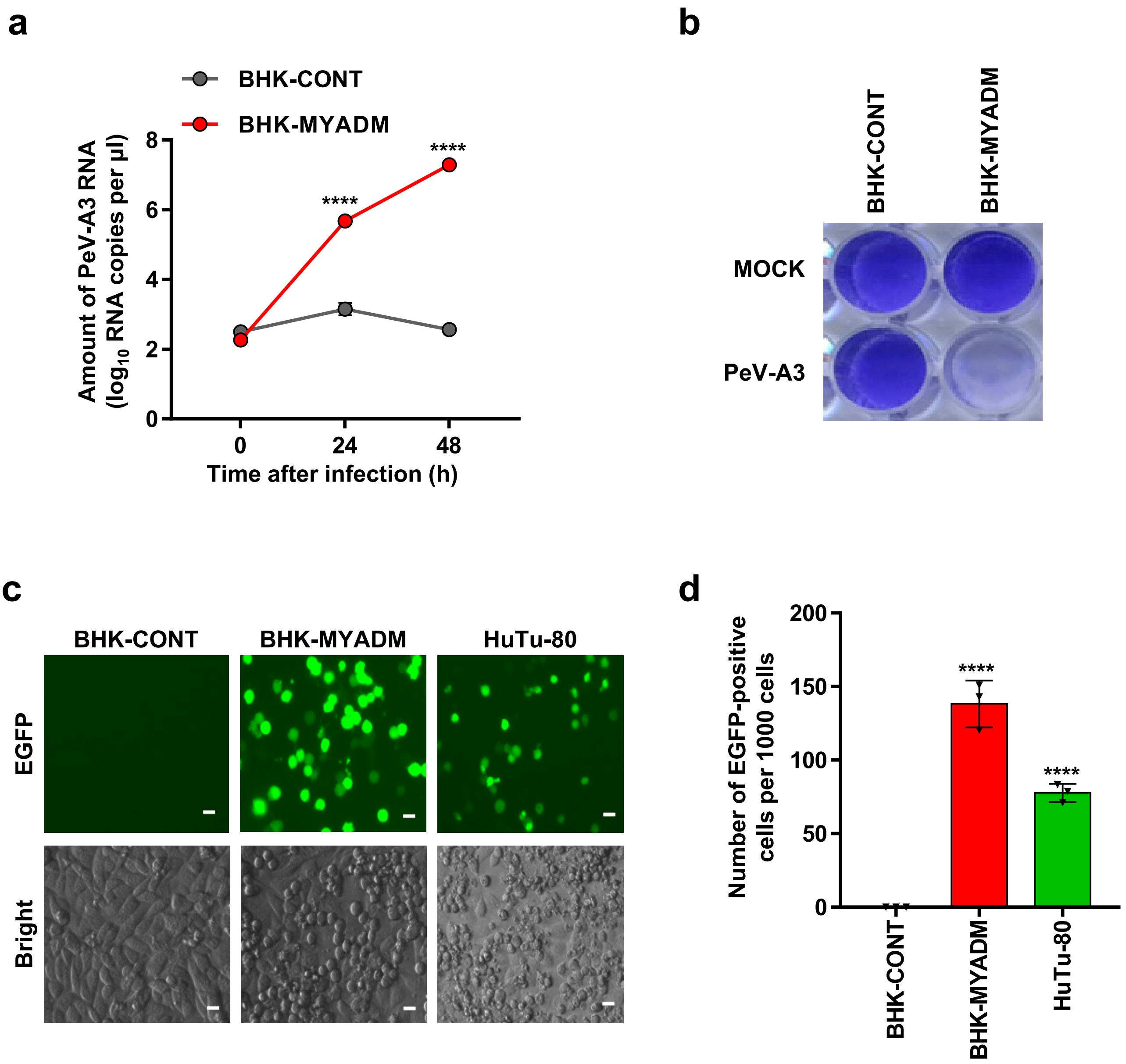

Supplementary Fig. 3 The expression of MYADM confers PeV-A3 infection to non-susceptible cells. a The amount of PeV-A3 (A308/99) RNA in the culture supernatant of BHK-CONT (dark gray circle) and BHK-MYADM (red circle) cells was determined by RT–qPCR, and the amount ($\log_{10}$ RNA copies per µl) is shown as the mean ± s.d. from triplicate experiments. Data from one experiment representative of two independent experiments (N = 2) are shown. P values were calculated by a two-way ANOVA with Sidak's multiple comparisons test. ****$P < 0.0001$. **b** The indicated cells in the 48-well plate were infected with PeV-A3 (A308/99), and the surviving cells that adhered to the plate were stained with crystal violet. Representative CPE data of two independent experiments (N = 2) are shown; the two experiments gave similar results. **c, d** The indicated cells were infected with PeV-A3-EGFP. At 24 h after infection, the number of EGFP-positive cells was determined by fluorescence microscopy and the ImageJ software program, and the number is shown as the mean ± s.d. from triplicate experiments. Data from one experiment representative of two independent experiments (N = 2) are shown. Scale bars, 20 µm (**c**). P values were calculated by a one-way ANOVA with Tukey's multiple comparisons test. ****$P < 0.0001$ (**d**). Source data are provided as a Source Data file.

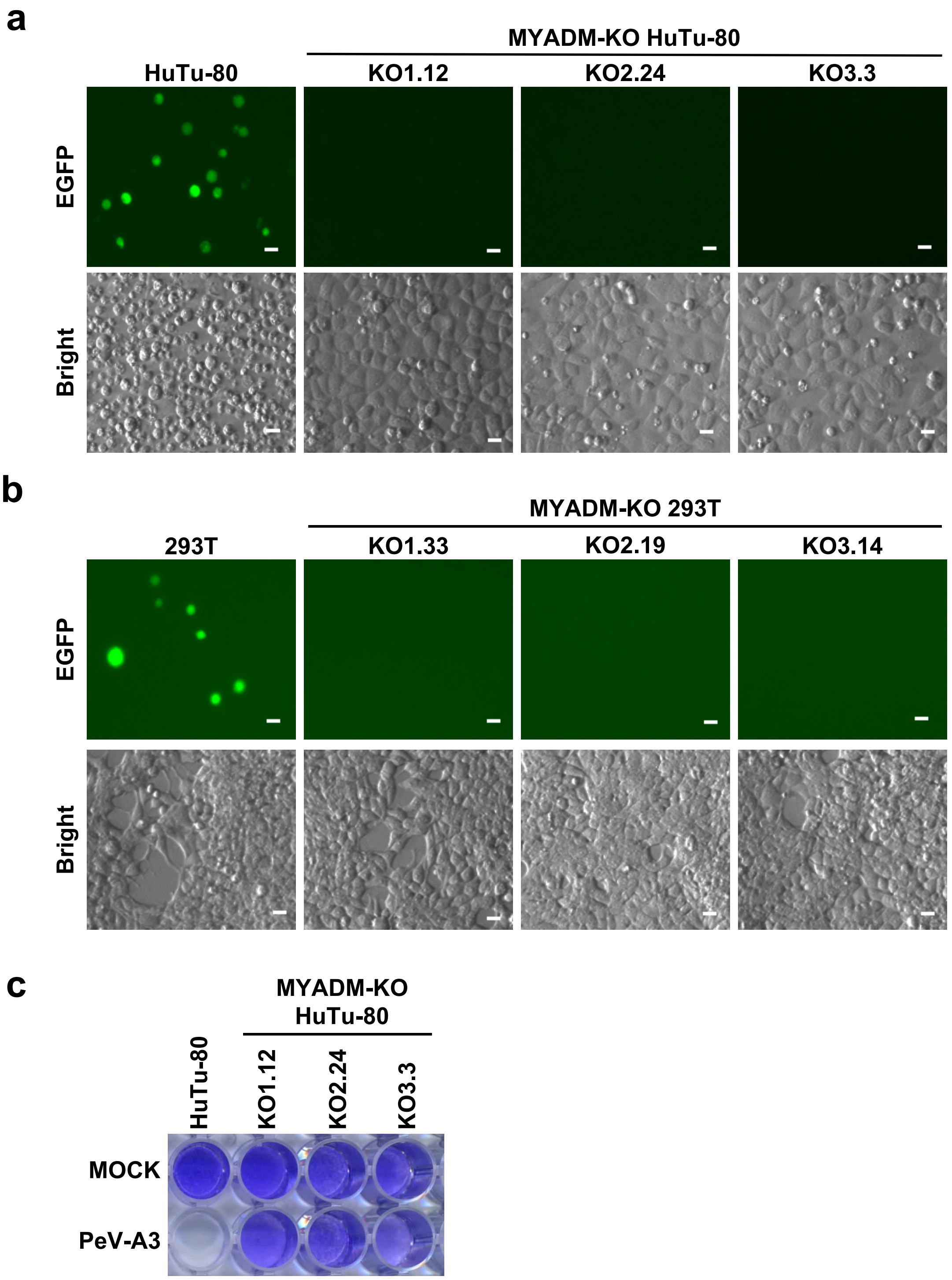

Supplementary Fig. 4 MYADM-KO abrogates PeV-A3 infection of susceptible cells. **a** HuTu-80 cells and the indicated MYADM-KO cells were infected with PeV-A3-EGFP. At 24 h after infection, the number of EGFP-positive cells per 1000 cells (Fig. 3e) was determined by fluorescence microscopy. Representative micrographs of two independent experiments (*N* = 2) are shown; the two experiments gave similar results. **b** 293T and the indicated MYADM-KO cells were infected with PeV-A3-EGFP. At 24 h after infection, the number of EGFP-positive cells per 1000 cells (Fig. 3f) was determined by fluorescence microscopy.

Representative micrographs of two independent experiments (N = 2) are shown; the two experiments gave similar results. Scale bars, 20 µm (**a, b**). **c** HuTu-80 and the indicated MYADM-KO cells in 48-well plates were infected with PeV-A3 (A308/99), and the cells that adhered to the plate were stained with crystal violet to evaluate CPEs. Representative CPE data of two independent experiments (N = 2) are shown; the two experiments gave similar results. Source data are provided as a Source Data file.

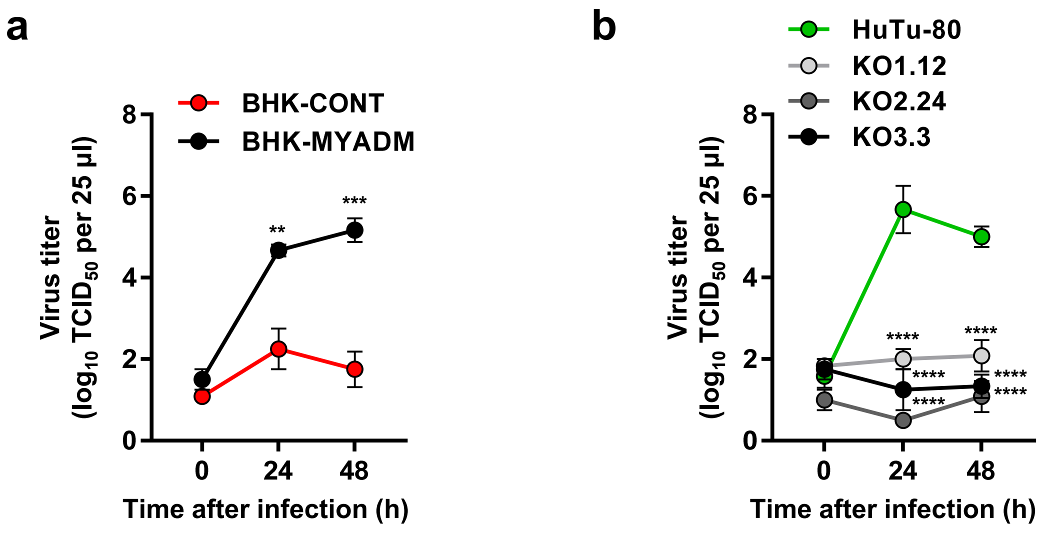

Supplementary Fig. 5 MYADM is essential for the infection of PeV-A3 clinical isolate. a BHK-MYADM (red circle) and BHK-CONT (black circle) cells were infected with Niigata-423/13 (PeV-A3 clinical isolate) at an MOI of 0.01, and the amount of virus (virus titer) in the culture supernatant was measured at 0, 24 and 48 h after infection. The virus titers in the culture supernatant are shown as the mean ± s.d. from triplicate experiments. Data from one experiment representative of two independent experiments (*N* = 2) are shown. *P* values were calculated by a two-way ANOVA with Sidak's multiple comparisons test. ***P* = 0.012. ****P* = 0.002. **b** HuTu-80 (green circle), MYADM-KO1.12 (light gray circle), KO2.24 (dark gray circle) and KO3.3 (black circle) cells were infected with Niigata-423/13 (PeV-A3 clinical isolate) at an MOI of 0.01, and the virus titer in the culture supernatant was measured at 0, 24 and 48 h after infection. The virus titers in the culture supernatant are shown as the mean ± s.d. from triplicate experiments. Data from one experiment representative of two independent experiments (*N* = 2) are shown. *P* values were calculated by a two-way ANOVA with Turkey's multiple comparisons test. *****P* < 0.0001. Source data are provided as a Source Data file.

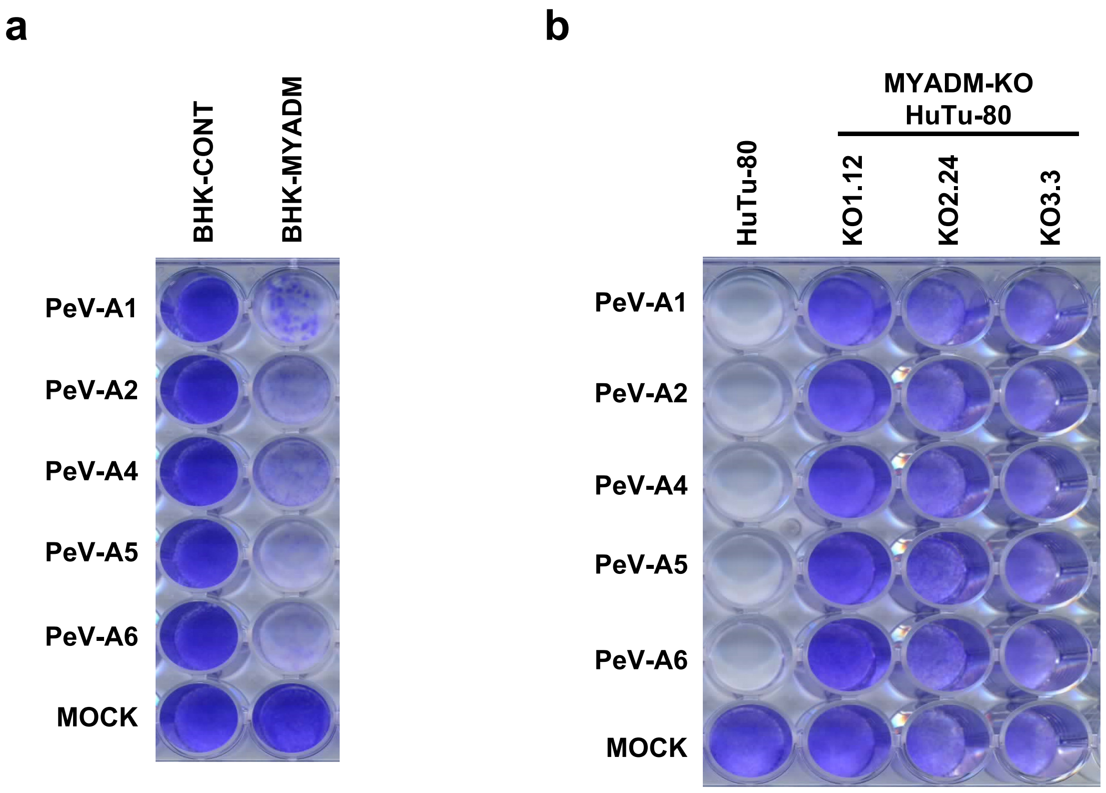

Supplementary Fig. 6 MYADM is essential for six PeV-A infection. a BHK-MYADM or BHK-CONT cells were infected with the indicated PeV-A genotypes, and then the cells were stained with crystal violet to evaluate CPEs. **b** HuTu-80 and the indicated MYADM-KO cells were infected with the indicated PeV-A genotypes, and the cells were stained with crystal violet to evaluate CPEs. Representative CPE data of two independent experiments ($N = 2$) are shown; the two experiments gave similar results (**a, b**). Source data are provided as a Source Data file.

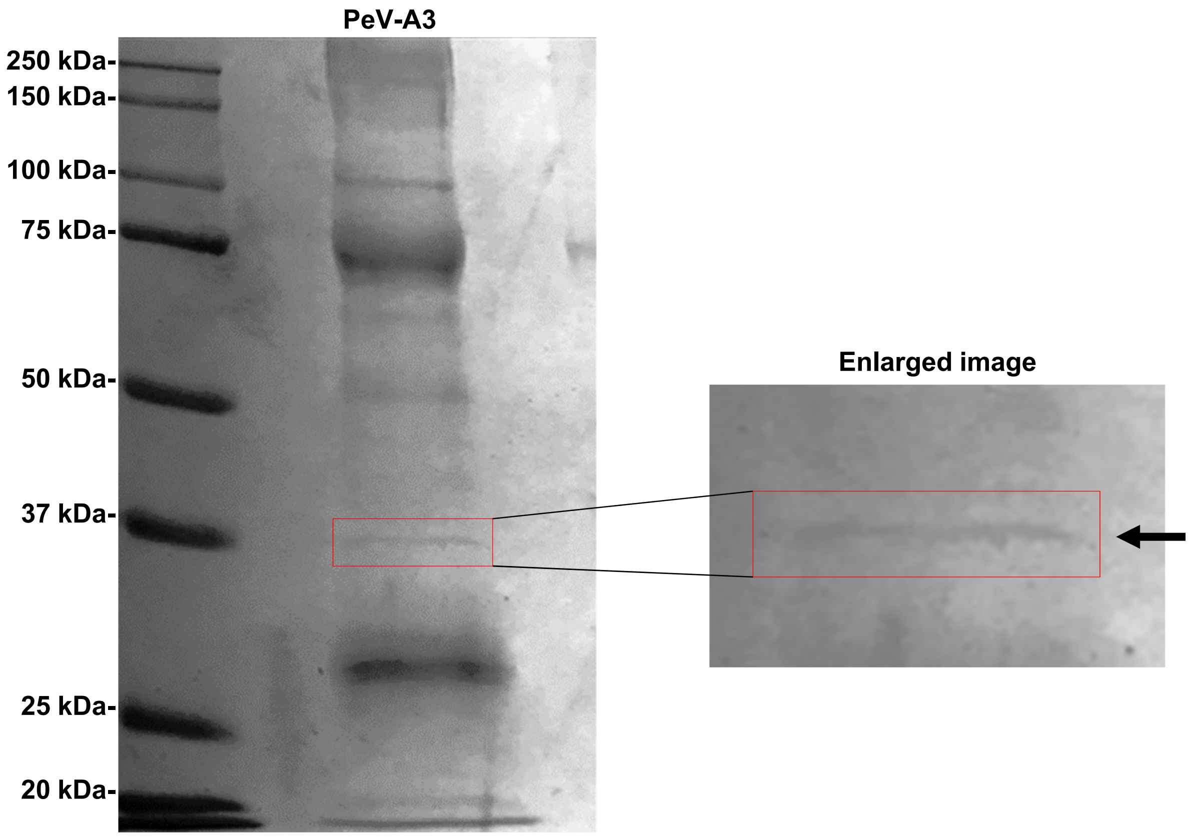

Supplementary Fig. 7 Mass spectrometric detection of PeV-A3 VP0 protein in the 35-kDa band of PeV-A3. PeV-A3 (Niigata-422/13) was purified from by cesium chloride density gradient centrifugation and separated by 10−15% SDS-PAGE, and the SDS-PAGE gel was stained with Coomassie Brilliant Blue (CBB). A gel containing the stained band around 35-kDa was cut out and analyzed using a TripleTOF5600+ mass spectrometer. SDS-PAGE experiment ($N = 1$) is shown. An arrow indicates the band that were cut out and analyzed by mass spectrometry. Source data are provided as a Source Data file.
