# Supplementary material for: Myeloid-associated differentiation marker is an essential host factor for human parechovirus PeV-A3 entry
Source: Nat Commun. 2023 Mar 31;14:1817. doi: 10.1038/s41467-023-37399-8 (PMC10066301; doi:10.1038/s41467-023-37399-8)
Supplement: Supplementary file 2 — Reporting Summary [file 41467_2023_37399_MOESM2_ESM.pdf]

nature portfolio

Corresponding author(s): Masahiro Fujii

Last updated by author(s): Mar 1, 2023

Reporting Summary

Nature Portfolio wishes to improve the reproducibility of the work that we publish. This form provides structure for consistency and transparency in reporting. For further information on Nature Portfolio policies, see our Editorial Policies and the Editorial Policy Checklist.

Statistics

For all statistical analyses, confirm that the following items are present in the figure legend, table legend, main text, or Methods section.

n/a	Confirmed	
☐	☒	The exact sample size (*n*) for each experimental group/condition, given as a discrete number and unit of measurement
☐	☒	A statement on whether measurements were taken from distinct samples or whether the same sample was measured repeatedly
☐	☒	The statistical test(s) used AND whether they are one- or two-sided<br>*Only common tests should be described solely by name; describe more complex techniques in the Methods section.*
☒	☐	A description of all covariates tested
☒	☐	A description of any assumptions or corrections, such as tests of normality and adjustment for multiple comparisons
☐	☒	A full description of the statistical parameters including central tendency (e.g. means) or other basic estimates (e.g. regression coefficient) AND variation (e.g. standard deviation) or associated estimates of uncertainty (e.g. confidence intervals)
☐	☒	For null hypothesis testing, the test statistic (e.g. *F*, *t*, *r*) with confidence intervals, effect sizes, degrees of freedom and *P* value noted<br>*Give P values as exact values whenever suitable.*
☒	☐	For Bayesian analysis, information on the choice of priors and Markov chain Monte Carlo settings
☒	☐	For hierarchical and complex designs, identification of the appropriate level for tests and full reporting of outcomes
☒	☐	Estimates of effect sizes (e.g. Cohen's *d*, Pearson's *r*), indicating how they were calculated

Our web collection on statistics for biologists contains articles on many of the points above.

Software and code

Policy information about availability of computer code

Data collection	No software was used.
Data analysis	Prism7 (GraphPad Software), Image J software version 1.53e (NIH), Mascot software version 2.6.0 (Matrix Science)

For manuscripts utilizing custom algorithms or software that are central to the research but not yet described in published literature, software must be made available to editors and reviewers. We strongly encourage code deposition in a community repository (e.g. GitHub). See the Nature Portfolio guidelines for submitting code & software for further information.

Data

Policy information about availability of data

All manuscripts must include a data availability statement. This statement should provide the following information, where applicable:

- Accession codes, unique identifiers, or web links for publicly available datasets
- A description of any restrictions on data availability
- For clinical datasets or third party data, please ensure that the statement adheres to our policy

The DNA sequences of PeV-A3-EGFP generated in this study are deposited in the DDBJ database under accession number LC723624.
DDBJ accession numbers are as follows: human MYADM, AY037147; macaque MYADM, XM_015124836; hamster MYADM, XM_005084151; rat MYADM, AY344060; and mouse MYADM, AK089538.
The relevant raw data generated in this study are provided in the Source data file.

Human research participants

Policy information about studies involving human research participants and Sex and Gender in Research.

Reporting on sex and gender	Not applicable
Population characteristics	Not applicable
Recruitment	Not applicable
Ethics oversight	Not applicable

Note that full information on the approval of the study protocol must also be provided in the manuscript.

Field-specific reporting

Please select the one below that is the best fit for your research. If you are not sure, read the appropriate sections before making your selection.

☒ Life sciences ☐ Behavioural & social sciences ☐ Ecological, evolutionary & environmental sciences

For a reference copy of the document with all sections, see nature.com/documents/nr-reporting-summary-flat.pdf

Life sciences study design

All studies must disclose on these points even when the disclosure is negative.

Sample size	Sample sizes were estimated on the basis of previous studies using similar methods, see Yamayoshi et al. (DOI: 10.1038/nm.1992); Zhao et al. (DOI: 10.1016/j.cell.2019.04.035).
Data exclusions	No data were excluded from the analyses.
Replication	All experiments except one experiment were repeated at least twice and were successful.
Randomization	Not relevant to this study, since samples were not allocated into experimental groups.
Blinding	Not relevant to this, since there are no group allocations in this study.

Reporting for specific materials, systems and methods

We require information from authors about some types of materials, experimental systems and methods used in many studies. Here, indicate whether each material, system or method listed is relevant to your study. If you are not sure if a list item applies to your research, read the appropriate section before selecting a response.

Materials & experimental systems

n/a	Involved in the study	
☐	☒	Antibodies
☐	☒	Eukaryotic cell lines
☒	☐	Palaeontology and archaeology
☒	☐	Animals and other organisms
☒	☐	Clinical data
☒	☐	Dual use research of concern

Methods

n/a	Involved in the study	
☒	☐	ChIP-seq
☒	☐	Flow cytometry
☒	☐	MRI-based neuroimaging

Antibodies

Antibodies used	rabbit polyclonal anti-MYADM (NBP2-24494, Novus), mouse monoclonal anti-β-actin (clone C4, sc-47778, Santa Cruz), anti-mouse IgG conjugated with horse radish peroxidase (HRP) (170-6516, Bio-Rad), anti-rabbit IgG conjugated with HRP (7074, Cell Signaling), mouse anti-FLAG antibodies (F1804, Merck), anti-mouse immunoglobulin labeled with Alexa488 (A-11029, Thermo Fisher Scientific), and polyclonal anti-PeV-A3 serum (anti-Niigata-422/13 serum) provided by Dr. Makoto Yamazaki, Denka Co. Ltd.
Validation	Commercially available antibodies were all validated by suppliers:<br>Rabbit polyclonal anti-MYADM (NBP2-24494, Novus) https://www.novusbio.com/products/myeloid-associated-differentiation-marker-antibody_nbp2-24494

Mouse monoclonal anti-β-actin (clone C4, sc-47778, Santa Cruz) https://www.scbt.com/p/beta-actin-antibody-c4?productCanUrl=beta-actin-antibody-c4&_requestid=2278161
Anti-mouse IgG conjugated with horse radish peroxidase (HRP) (170-6516, Bio-Rad) https://www.bio-rad.com/en-jp/sku/1706516-goat-anti-mouse-igg-h-l-hrp-conjugate?ID=1706516
Anti-rabbit IgG conjugated with HRP (7074, Cell Signaling) https://www.cellsignal.com/products/secondary-antibodies/anti-rabbit-igg-hrp-linked-antibody/7074?country=JP&language=en
Mouse anti-FLAG antibodies (F1804, Merck) https://www.sigmaaldrich.com/JP/en/product/sigma/f1804?gclid=EAIaIQobChMIq8L5_L2o_QIVBFVgCh0q3AnREAAYASAAEgIWrPD_BwE
Anti-mouse immunoglobulin labeled with Alexa488 (A-11029, Thermo Fisher Scientific) https://www.thermofisher.com/antibody/product/Goat-anti-Mouse-IgG-H-L-Highly-Cross-Adsorbed-Secondary-Antibody-Polyclonal/A-11029

Polyclonal anti-PeV-A3 serum (anti-Niigata-422/13 serum provided by Dr. Makoto Yamazaki, Denka Co. Ltd.) was validated by neutralization of PeV-A3 (A308/99 strain) by 50% Tissue Culture Infectious Dose neutralization assay.

Eukaryotic cell lines

Policy information about cell lines and Sex and Gender in Research

Cell line source(s)

HuTu-80 and SW620 cells were purchased from ATCC. HuTu-80: ATCC; HTB-40, SW620: ATCC; CCL-227.
293T, C33A, Saos2, and Jurkat cells; Takahashi et al, Mol Cell Biol, 2013. 293T: ATCC; CRL-3216, C33A: ATCC; HTB-31, Saos2: ATCC; HTB-85, Jurkat: ATCC; TIB-152.
HeLa cells; Kakihana et al, iScience, 2021. HeLa: ATCC; CCL-2.
SH-SY5Y cells; Sango et al, J Biol Chem, 2022. SH-SY5Y: ATCC; CRL-2266.
BHK-21 cells; Taniura et al, J Virol, 2009. BHK-21: ATCC; CCL-10.
LLC-MK2 cells; Watanabe et al, Emerg Infect Dis, 2007. LLC-MK2: ATCC; CCL-7.
NIH/3T3 cells; Koi et al, Microbiol Immunol, 1981. NIH/3T3: ATCC; CRL-1658.

Authentication

Cell lines were not authenticated.

Mycoplasma contamination

All cell lines used in this study were tested negative for mycoplasma contamination.

Commonly misidentified lines
(See ICLAC register)

No commonly misidentified cell lines were used.
